# Biosynthesis of Fusapyrone Depends on the H3K9 Methyltransferase, FmKmt1, in *Fusarium mangiferae*

**DOI:** 10.3389/ffunb.2021.671796

**Published:** 2021-07-06

**Authors:** Anna K. Atanasoff-Kardjalieff, Friederike Lünne, Svetlana Kalinina, Joseph Strauss, Hans-Ulrich Humpf, Lena Studt

**Affiliations:** ^1^Department of Applied Genetics and Cell Biology, Institute of Microbial Genetics, University of Natural Resources and Life Sciences, Vienna (BOKU), Tulln an der Donau, Austria; ^2^Institute of Food Chemistry, Westfälische Wilhelms-Universität, Münster, Germany

**Keywords:** heterochromatin, histone PTMs, *Fusarium mangiferae*, *Fusarium fujikuroi* species complex, H3K9me3, secondary metabolism, fusapyrone, deoxyfusapyrone

## Abstract

The phytopathogenic fungus *Fusarium mangiferae* belongs to the *Fusarium fujikuroi* species complex (FFSC). Members of this group cause a wide spectrum of devastating diseases on diverse agricultural crops. *F. mangiferae* is the causal agent of the mango malformation disease (MMD) and as such detrimental for agriculture in the southern hemisphere. During plant infection, the fungus produces a plethora of bioactive secondary metabolites (SMs), which most often lead to severe adverse defects on plants health. Changes in chromatin structure achieved by posttranslational modifications (PTM) of histones play a key role in regulation of fungal SM biosynthesis. Posttranslational tri-methylation of histone 3 lysine 9 (H3K9me3) is considered a hallmark of heterochromatin and established by the SET-domain protein Kmt1. Here, we show that FmKmt1 is involved in H3K9me3 in *F. mangiferae*. Loss of FmKmt1 only slightly though significantly affected fungal hyphal growth and stress response and is required for wild type-like conidiation. While FmKmt1 is largely dispensable for the biosynthesis of most known SMs, removal of *FmKMT1* resulted in an almost complete loss of fusapyrone and deoxyfusapyrone, γ-pyrones previously only known from *Fusarium semitectum*. Here, we identified the polyketide synthase (PKS) FmPKS40 to be involved in fusapyrone biosynthesis, delineate putative cluster borders by co-expression studies and provide insights into its regulation.

## Introduction

*Fusarium* is a species-rich genus of filamentous fungi that collectively can cause agriculturally significant diseases on virtually all crop plants (Leslie and Summerell, 2006). During growth and infection, these fungi produce small molecular weight compounds, also referred to as secondary metabolites (SMs), including harmful mycotoxins that are of concern to food and feed safety (Munkvold, 2017). *Fusarium*-induced crop diseases and mycotoxin contaminations result in significant economic losses to world agriculture every year (Wu, 2007). Phytopathogenic fungi often produce a specific set of SMs during the fungal-host interaction, which allows them to overcome the plant's natural defense mechanisms (Brown and Proctor, 2013; Streit et al., 2013; Perincherry et al., 2019). A central subgroup of the genus *Fusarium* belongs to the *Fusarium fujikuroi* species complex (FFSC), including several detrimental species in agriculture (Niehaus et al., 2017). A yet only poorly characterized member of the FFSC is *Fusarium mangiferae*. The ascomycete fungus causes the notorious Mango Malformation Disease (MMD) (Summanwar et al., 1966; Varma et al., 1974; Ibrahim et al., 1975) on mango (*Mangifera indica*) trees in tropical and subtropical areas. Infected plants are often recognized by vegetative malformation on seedlings or young trees (Freeman et al., 2014). Infection of vegetative tissue often leads to symptoms such as the malformation of shoots from apical and auxiliary buds resulting in a “bunchy-top” appearance, combined with shortened internodes and dwarfed and narrowed leaves (Newman et al., 2012; Freeman et al., 2014). Noteworthy, affected seedlings will most often remain stunted and do not survive such an early fungal invasion (Varma et al., 1974). In contrast, floral malformation will result in shortened, thickened and highly branched panicles with increased flower number and size (Freeman et al., 2014). Infected inflorescence tissue then is prone to either form sterile panicles or abort fruit production shortly after onset (Varma et al., 1974; Freeman et al., 2014), resulting in crop reduction of 50–60% and in severe cases to the complete loss of harvest (Chakrabarti, 2011; Ansari et al., 2015). MMD symptoms likely result from the production of SMs including plant hormones, such as cytokinins, that are associated with the abnormal plant development during mango malformation (Stadent and van Nicholson, 1989; Stadent et al., 1989; Vrabka et al., 2019). Next to this, recent studies performed on inflorescence and vegetative tissues infected with *F. mangiferae* suggests that the fungus affects the plant's hormonal balance by targeted up- and downregulation of important plant growth regulators such as gibberellins or auxins (Usha et al., 2019). Apart from the plant hormones, only little is known about the chemical potential of *F. mangiferae*. Recent comparative genomic studies of *F. mangiferae* revealed a total of 52 putative and yet mostly cryptic SM key enzyme-encoding genes (Niehaus et al., 2016a).

SMs are thought to provide selective advantages for the producing organism under defined conditions (Macheleidt et al., 2016). This implies a tight regulation of the involved genes. SM biosynthetic genes are often clustered in the fungal genome (Keller and Hohn, 1997). Here, co-regulation of genes may be facilitated by chromatin-based mechanisms (Strauss and Reyes-dominguez, 2011; Collemare and Seidl, 2019; Pfannenstiel and Keller, 2019). In eukaryotes, all DNA-dependent processes including SM gene expression depend on the chromatin structure. Chromatin is the fundamental packaging form of DNA, which is wrapped around an octamer of canonical core histones H2A, H2B, H3, and H4. This structure generates a natural obstacle to the transcription machinery and thus must be highly dynamic and well-coordinated upon (environmental or developmental) stimuli. Alterations in the chromatin structure involve energy-dependent nucleosome rearrangements, the exchange of core histones with their respective variants (Chen and Ponts, 2020) and post-translational modifications (PTMs) of histones (Brosch et al., 2008). Histone PTMs such as acetylation, methylation or phosphorylation take place on distinct amino acid residues, thereby altering the chromatin structure and consequently the accessibility of the underlying DNA. Next to this, decorating histone PTMs provide a recognition platform for other chromatin-modifying enzymes, remodeling complexes and transcription factors, which consequently regulate transcription of underlying genes (Bannister and Kouzarides, 2011). Tri-methylation of histone 3 lysine 9 (H3K9me3) is considered a hallmark of constitutive heterochromatin, and mainly found at centromeric and telomeric gene-poor regions from fission yeast to mammals (Craig, 2005; Smith et al., 2008; Becker et al., 2016). Generally, H3K9me3 is associated with essential functions regarding coordinated gene silencing and genome defense against parasitic elements in higher eukaryotes (Henikoff, 2000; Selker, 2004). The involved SET [(Su(var)3-9), enhancer of zeste [E(z)] and trithorax] domain-containing histone methyltransferase was first identified in the model organism *Drosophila melanogaster* (Tschiersch et al., 1994). Later, homologs were identified and characterized in diverse model organisms including *Schizosaccharomyces pombe* (*SpClr4*) (Nakayama et al., 2001), *Aspergillus nidulans* (*clrD*) (Reyes-Dominguez et al., 2010) and *Neurospora crassa* (*NcDIM-5*) (Tamaru and Selker, 2001). In *N. crassa, DIM-5* deletion led to some growth abnormalities and is essential to establish DNA methylation patterns in this species (Tamaru and Selker, 2001; Freitag et al., 2004). Notably, studies in various filamentous fungi suggest species-specific roles for DIM-5 homologs, with varying degrees of phenotypic appearances. A large body of research was dedicated to study the importance of H3K9me3 in regard to fungal development, but less is known about its influence on fungal SM production. For *A. nidulans*, clrD negatively influences the expression of the sterigmatocystin cluster genes *aflR* (pathway-specific activator gene) and *stcO*, resulting in elevated sterigmatocystin production under SM-inducing conditions (Reyes-Dominguez et al., 2010). Similarly, for *E. festucae*, lack of the NcDIM-5 homolog EfclrD resulted in a de-repression of lolitrem and ergot alkaloid cluster genes in axenic culture, normally solely expressed under symbiotic conditions (Chujo and Scott, 2014). In the case of *F. verticillioides*, lack of FvDim5 resulted in an increased production of a melanin-like pigment, suggesting that FvDim5 is involved in SM production also in this fungus (Gu et al., 2017).

Here, we focus on the functional homolog of the well-characterized H3K9-specific histone methyltransferase NcDIM-5, FmKmt1, involved in H3K9me3 in the phytopathogenic ascomycete *F. mangiferae*. Loss of FmKmt1 had a moderate though significant impact on fungal hyphal growth and asexual development. SMs known to be produced by members of the FFSC (Niehaus et al., 2016a) remained largely unaffected by the loss of FmKmt1, but deletion of *FmKMT1* resulted in significantly decreased amounts of fusapyrone (FPY) and deoxyfusapyrone (dFPY), γ-pyrones previously only known for *Fusarium semitectum* (synonym *Fusarium incarnatum*; Evidente et al., 1994; Hiramatsu et al., 2006). Both SMs were produced in high amounts by the wild-type strain under defined conditions, while they were almost completely abolished in Δ*fmkmt1* strains. This suggests that a functional FmKmt1 is crucial for FPY biosynthesis in this species. In this work, we used a combination of bioinformatics, reverse genetics and co-expression studies to identify the responsible key enzyme involved in FPY biosynthesis (FmPKS40), determined putative FPY cluster genes (*FmFPY1*-*FmFPY7*) and studied *FmFPY* gene regulation in *F. mangiferae* and other members of the FFSC.

## Materials and Methods

### Fungal Strains, Media, and Growth Conditions

The *F. mangiferae* MRC7560 wild-type strain (FmWT) originating in Israel (National Research Institute for Nutritional Diseases, Tygerberg, South Africa) was used as parental strain for the generation of deletion (Δ*fmkmt1*, Δ*fmppt1*), *ex situ* complementation (Δ*fmkmt1*/*FmKMT1*^Ces^), *FmPKS8* partial gene deletion (Δ*fmPKS8*), *FmPKS40* partial gene deletion (Δ*fmPKS40*) and amino acid exchange of histone 3 (H3) lysine 9 (K9) to arginine 9 (R9) (FmH3K9R). For gene expression studies the wild-type strains *F. verticillioides* M3125 (D. Brown from the U.S. Department of Agriculture, U.S.A); *Fusarium proliferatum* ET1 (Elena Tsavkelova, Moscow State University, Russia), and *F. proliferatum* NRRL62905 (Robert Proctor from the U.S. Department of Agriculture, U.S.A) were used. For SM and expression analysis, the *Fusarium fujikuroi* wild-type strain B14 was kindly provided by S.-H. Yun, Korea.

For genomic DNA (gDNA) and RNA isolation, all strains were grown for 2–3 days on solid complete media (CM) (Pontecorvo et al., 1953). Plates were covered with cellophane sheets (Folia Bringmann) and incubated at 30°C in the dark. Fungal growth assays were performed on solid CM, V8 (30 mM CaCO_3_, 20%, v/v, vegetable juice; Campbell Food, Puurs, Belgium), *Fusarium* minimal medium (FMM) (Correl et al., 1987) and synthetic ICI (Geissman et al., 1966) medium supplemented with 6 mM sodium nitrate (NaNO_3_, Carl Roth). Plates were inoculated with 10 μL of a 10^5^ conidia/mL solution and incubated at 30°C in the dark for 5 days. Stress tests were performed on solid CM. For the assessment of osmotic stress, plates were supplemented with either 1 M sorbitol (Sigma-Aldrich) or 1 M sodium chloride (NaCl, Sigma-Aldrich). The plates were inoculated with a 5 mm agar plug each and incubated for 7 days at 30°C under dark conditions. Conidiospore production was assessed on solid V8. Medium was inoculated with a 5 mm agar plug each and incubated at 20°C for 7 days under the presence of 18 h light and 6 h dark (L/D) or dark conditions (D) and 70% humidity. Conidia were counted using a Fuchs-Rosenthal hemocytometer under a light microscope (Carl Zeiss). For single spore isolation, microconidia production was induced on solid V8 under dark conditions at 30°C for 3–5 days. Subsequently, harvested conidia were plated on solid CM with respective selection marker and plates were incubated for 1–2 days at 30°C in the dark. Resulting microconidia generally harbor only one nucleus each, thus, resulting in homokaryotic strains. For fungal liquid cultivations, mycelia were pre-cultured in 100 mL Darken medium (Darken et al., 1959) in a 300 mL Erlenmeyer flask for 3 days in the dark (30°C, 180 rpm). Then, 0.5 mL of the pre-culture was transferred to ICI media supplemented with either 6 or 60 mM glutamine (Carl Roth) or with 6 or 120 mM NaNO_3_ as sole nitrogen source and incubated on an orbital shaker at 180 rpm, 30°C. Mycelia were harvested after 3–7 or 7 days for RNA or SM analysis, respectively. In the case of protein extractions, mycelia was grown in liquid ICI with 120 mM NaNO_3_ and harvested 4 days post inoculation.

### Plasmid Construction

For the construction of (partial) deletion, complementation and site-directed mutagenesis vectors, yeast recombination cloning was performed, as described earlier (Colot et al., 2006; Schumacher, 2012). All primers were obtained from Sigma-Aldrich and are listed in Supplementary Table 1. PCR products were amplified using a high-fidelity polymerase (Q5-polymerase, New England Biolabs or Phusion High-Fidelity DNA polymerase, Thermo Scientific). Briefly, for the generation of targeted gene deletion vectors ~1 kb upstream (5′) and downstream (3′) of the gene of interest were amplified from FmWT gDNA with the primer pairs 5F/5R and 3F/3R. For gene deletion constructs, the hygR resistance cassette was amplified from the template vector pCSN44 (Staben et al., 1989) with the primer pair hphF/hphR. In the case of partial gene deletion, the first 2 kb of the wild-type gene was replaced by the hygR resistance cassette. Fragment generation was performed by amplification of 1 kb upstream (5′) of the gene of interest using the primers 5F//5R. The 3' region was amplified from 1 kb of the native wild-type gene using primers disr3F//disr3R. For generation of the Δ*fmkmt1*/*FmKMT1* complementation vector, *FmKMT1* driven by its native promoter was amplified from *F. mangiferae* gDNA with primers FmKMT1_5F//FmCIL_tgluc, followed by the amplification of the Tgluc terminator sequence from *Botrytis cinerea* B05.10 (BcTgluc) with the primer pair TglucF2//Tglucnat1R (Studt et al., 2017). Next, amplification of genR resistance cassette from a template plasmid (pΔ*fgkdm5*/kdmB^Cis^) containing the geneticin resistance cassette (Bachleitner et al., 2019) was performed with the primer pair GeniTglucR//GeniF, and amplification of the 3′ region with the primers FmKmt1_3F/3R (Supplementary Figure 2A). For amino acid exchange at H3K9 (H3K9R), the *H3* gene was amplified together with 1 kb upstream region with primer pairs Histone3_Mut_1F//Histone3_Mut_K9R_1R and Histone3_Mut_K9R_2F//Histone3_Mut_2R. The gene was followed by BcTgluc and hygR amplified with the primer pairs Tgluc_F2//Tgluc-nat1-R and hphR//hphF using genomic DNA of *B. cinerea* B05.10 and pCSN44, respectively, as templates. The downstream region of *H3* was amplified with the primer pair Histone3_Mut_4F//Histone3_Mut_4R (Supplementary Figure 5). *Saccharomyces cerevisiae* FY834 was transformed with respective PCR fragments, resistance cassettes and the *Eco*RI/*Xho*I-digested shuttle vector pRS426 (Christianson et al., 1992).

### Fungal Transformation

*F. mangiferae* protoplasts generation was performed as described elsewhere (Tudzynski et al., 1999). Linear gene replacement or partial gene deletion cassettes were amplified from the circular deletion vectors, pΔ*fmkmt1*, pΔ*fmppt1*, and pΔ*fmPKS8* with the appropriate primer pairs 5F//3R (Supplementary Table 1). Since the number of successful homologous recombination events were low, the split marker approach (Goswami, 2012) was performed for the targeted partial gene deletion of *FmPKS40*. For this, fragments were amplified from pΔ*fmPKS40* by 5F//Split-mark_hphF and 3R//Split-mark_hphR (Supplementary Table 1). All fragments used for subsequent transformation were generated using a proof-reading polymerase (Q5-polymerase, New England Biolabs or Phusion Flash High-Fidelity PCR Master Mix, Thermo Fisher Scientific). For the complementation of Δ*fmkmt1*, 10 μg of *Pvu*I/*Vsp*I-digested pΔ*fmkmt1*/*FmKMT1*^Ces^ was used (Supplementary Figure 2A). For the generation of the FmH3K9R strain, 10 μg of pH3K9R was digested with *Pvu*II and used for subsequent fungal transformation (Supplementary Figure 5B). Fungal transformants were selected on regeneration media supplemented with either 100 ppm hygromycin B (Merck Millipore) or 100 ppm geneticin (Fermtech Garching), depending on the resistance marker. Successful homologous integration events and if applicable absence of respective wild-type genes were verified by diagnostic polymerase chain reaction (PCR). Successful complementation of Δ*fmkmt1* was additionally verified by reverse transcriptase-quantitative PCR (RT-qPCR) analysis. Insertion of the desired point mutation (FmH3K9R) was additionally verified by sequencing (Supplementary Figure 5A).

### Standard Molecular Techniques

Genomic DNA (gDNA) was extracted from ground and lyophilized mycelia as described earlier (Cenis, 1992). To verify *in situ* integration events of (partial) deletions as well as *ex situ* integration of the complementation construct, diagnostic PCRs were performed with GoTaq^®^ G2 DNA Polymerase (Promega). For deletion constructs, the respective primer pairs dia5F//pCSN44_trpCT and dia3F//pCSN44_trpcP2 were used. For partial gene deletion, the primers dia5F//pCSN44_trpCT and dia3R//diaWT_R were used. Absence of the wild-type gene was verified with the respective WT_F//R primers. Following primer pairs were used for verification of the complementation construct: for the presence of the geneticin cassette genseq3//genR_split_F primers were used and presence of the wild-type gene was verified with FmKMT1_diaWT_F//R primers. Cultivations of *S. cerevisiae* and *Escherichia coli* were performed as described elsewhere (Schumacher, 2012). Yeast pDNA was extracted with the GeneJET Plasmid Miniprep Kit (Thermo Fisher Scientific) and directly used as template for the amplification of gene replacement and partial gene deletion cassettes. Transformation of the complementation and FmH3K9R constructs in *E. coli* DH5α (Invitrogen™) was performed according to the manufacturer's procedure. Subsequent extraction of pDNA was performed with the GeneJET Plasmid Miniprep Kit (Thermo Fisher Scientific) as described in the manufactures protocol. Correctness of the complementation and FmH3K9R vectors was confirmed by sequencing (LGC Genomics, Germany). All primers used for sequencing are listed in Supplementary Table 1. For expression analysis, lyophilized and ground mycelium from either fungal cultivation on CM or cultivation under SM-producing standard conditions (ICI with 6 or 120 mM NaNO_3_, 6 or 60 mM glutamine) for 4–7 days was used for RNA isolation with the RNA reagent TRIzol (Thermo Fisher Scientific) according to the manufacturer's instructions.

### Western Blot Analysis

For total protein extraction, lyophilized mycelia of FmWT, Δ*fmkmt1*, Δ*fmkmt1*/*FmKMT1*^Ces^ and FmH3K9R were used. Proteins were extracted as described earlier (Studt et al., 2016b). For western blot analysis roughly 30 or 50 μg of total proteins were applied and the membrane was probed with the following primary antibodies: anti-H3 C-Term antibody (Active motif AM39766), anti-H3K9me3 (Abcam, ab8898/Active motif, AM39161), anti-H3K9ac (Abcam, ab4441). Anti-rabbit HRP conjugated secondary antibody (Jackson ImmunoResearch) was applied to all primary antibodies. For detection of chemiluminescence signal, the western membrane was developed with Clarity™ ECL Western Substrate and visualized with a ChemiDoc™ XRS (Bio-Rad) system. Subsequent densitometric analyses of western blot signals were performed using the ImageJ software. Signals were normalized to the histone H3 C-term signal and the wild-type signal ratio was set to 100% for referencing.

### Expression Analysis by Semi-Quantitative PCR and Quantitative RT-qPCR

For cDNA synthesis, 1 μg RNA was treated with DNase I (Thermo Fisher Scientific) and subsequently transcribed into cDNA with either iScript™ cDNA Synthesis Kit (Bio-Rad) or LunaScript™ RT SuperMix Kit (NEB). For semi-quantitative PCR, cDNA was used as template to perform PCR analysis using GoTaq^®^ G2 DNA Polymerase. Fragment amplification was then performed with primers also designed for RT-qPCR and 30 cycles per run. For cDNA quantity comparison, the house-keeping gene actin (FMAN_05925) was utilized and amplified with the primer pair Actin_F//R. For RT-qPCR analysis the iTaq™ Universal SYBR^®^ Green Supermix (Bio-Rad) was applied and an iCycler iQ Real-Time PCR System (Bio-Rad) was used for quantification. In all cases the primer efficiency was kept between 90 and 110%, Ct values greater than 31 were taken as not expressed. Results were calculated according to the ΔΔCt (Pfaffl, 2001). Expression of all tested genes were normalized to the expression of actin (FMAN_05925), Glyceraldehyde-3-phosphate dehydrogenase (GPD, FMAN_05925), and β-tubulin (FMAN_07563). All primers used for semi-quantitative as well as RT-qPCR are listed in Supplementary Table 1.

### SM Production and Chemical Analysis

Supernatant used for chemical analysis of the respective fungal strains was retrieved from 7-day-old liquid ICI cultures supplemented with different nitrogen sources. The supernatants were filtered through the 0.2 μm syringe filters (Nylon membrane, 1.3 mm, non-sterile, Agilent, Waldbronn, Germany). An aliquot of 400 μL filtrate was diluted 1:1 with acetonitrile (MeCN), the samples were filtered a second time, and directly used for screening analysis. FPY and dFPY analytical standards (purity ≥ 96%) were purchased from Santa Cruz Biotechnology (Dallas, TX, USA) and Cayman Chemical (Ann Arbor, MI, USA), respectively. For quantitation of FPY and dFPY, samples were diluted accordingly. The screening of the metabolites and quantitation analysis of FPY and dFPY were performed on a Nexera XR LC-system (Shimadzu, Duisburg, Germany) coupled to a Fourier Transform Mass Spectrometer (FTMS) with a heated electrospray ionization (ESI) source operated in positive ionization mode (LTQ-Orbitrap XL, Thermo Fisher Scientific, Bremen, Germany). Chromatographic separation was performed on a Nucleodur^®^ Phenyl-Hexyl column (100 ^*^ 2 mm i.d., particle size 3 μm) equipped with a 4 ^*^ 2 mm i.d. Phenyl-Hexyl guard column (Macherey-Nagel, Düren, Germany). For the screening method the gradient started with 5% MeCN + 0.1% formic acid (FA) (solvent A) and 95% H_2_O + 0.1% FA (solvent B) for 2 min and a flow rate of 0.4 mL/min. Within 24 min, A was raised to 95%. For the next 5 min, A was held at 95%. Equilibration was performed for 4 min. For quantitation of FPY and dFPY, the gradient started at 30% of A with a flow rate of 0.35 mL/min, rising to 95% of A in 8.5 min. These conditions were held for 2 min, followed by an equilibration step at 30% of A for 3.1 min. The calibration was performed in a range of 50–500 ng/mL FPY and dFPY. For the screening method and quantitative determination of FPY and dFPY, ESI was applied with a source voltage set to +4 kV and capillary and vaporizer temperatures of 350°C. The sheath gas flow was set to 40 arbitrary units, auxiliary gas flow to 20 arbitrary units, and sweep gas flow to 10 arbitrary units. Xcalibur, Version 3.1.66.10 Tune Plus (Thermo Fisher Scientific, Dreieich, Germany) software was used for data acquisition and analysis.

## Results

### FmKmt1 Is a Histone H3 Lysine 9 (H3K9)-Specific Methyltransferase in *Fusarium mangiferae*

**Figure 1:**
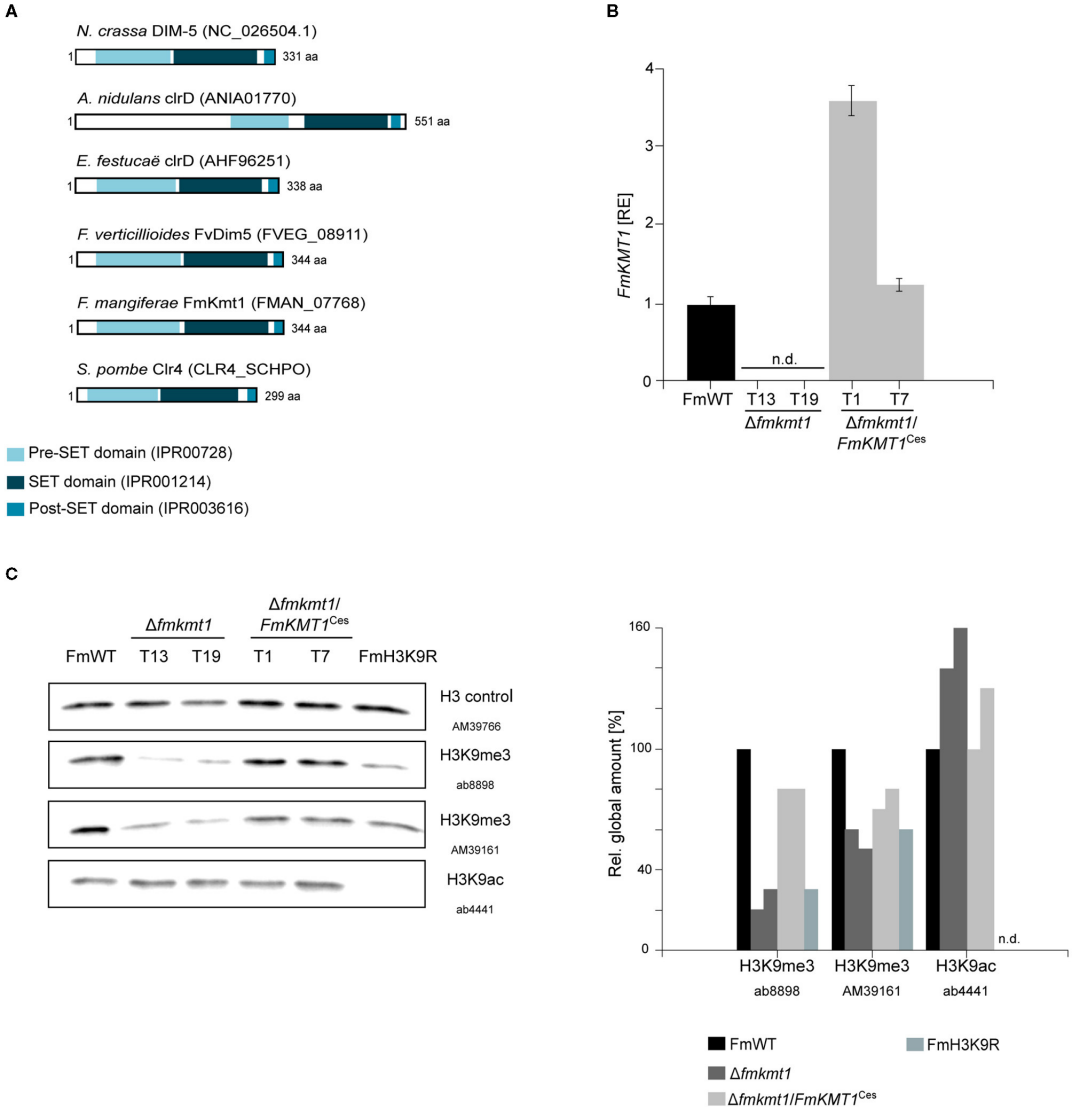
FmKmt1 is involved in H3K9me3 in *F. mangiferae*. **(A)** Graphical representation of the domain structure of *Neurospora crassa* DIM-5 and orthologs in other fungal species including *F. mangiferae* (FmWT). The conserved pre-Set domain, SET domain, and post-SET domain are indicated; InterPro accession numbers are shown in the domain description **(B)** For *FmKMT1* expression analysis, FmWT, Δ*fmkmt1*, and Δ*fmkmt1*/*FmKMT1*^Ces^ were grown on solid CM for 3 days at 30°C. For the determination of transcript levels, RNA was extracted and transcribed into cDNA prior to RT-qPCR. The FmWT *FmKMT1* expression was arbitrarily set to 1. Mean values and standard deviations are shown. n.d., not detected; RE, relative expression. **(C)** Western blot analysis of FmWT, Δ*fmkmt1*, Δ*fmkmt1*/*FmKMT1*^Ces^, and FmH3K9R using two anti-H3K9me3-specific antibodies (AB, Abcam; AM, Active Motif), and one anti-H3K9ac-specific antibody. For referencing one anti-H3-specific antibody was used. Indicated strains were grown in liquid ICI medium supplemented with 120 mM NaNO_3_ for 4 days and subsequently total proteins were extracted. Roughly, 50 μg of total protein extracts were separated on a SDS gel prior to western blotting. For quantification, a densitometric analysis was performed and the respective wild-type strain was arbitrarily set to 100%. n.d., not detected *via* Image J analysis tool.

The putative H3K9-specific methyltransferase in *F. mangiferae*, FmKmt1, was identified by the orthology prediction program QuartetS (Yu et al., 2011). Function- and phylogeny-based metrics based on large-scale comparison predicted the protein, FMAN_07768 (FmKmt1) as the true ortholog of the H3K9-specific methyltransferase NcDIM-5 (NCU04402) in *N. crassa* (Tamaru and Selker, 2001). Pairwise sequence alignment using LALIGN (Huang and Miller, 1991) showed 56.3% sequence identity (*E*-value 6.9 × 10^−110^) on the protein level between FmKmt1 and NcDIM-5. FmKmt1 is predicted to contain the catalytically active SET [Su(var)3-9, Enhancer-of zeste and Trithorax] domain including a pre- and post-SET domain, flanking the actual SET domain. This domain architecture is known from other orthologs to be required for full functionality of Kmt1 (Figure 1A) (Dillon et al., 2005). Orthologs of Kmt1 have also been studied in other fungi including *A. nidulans* clrD (Reyes-Dominguez et al., 2010), *E. festucae* clrD (Chujo and Scott, 2014), *B. cinerea* BcDim5 (Zhang et al., 2016) as well as the FFSC member *F. verticillioides* FvDim5 (Gu et al., 2017). No *KMT1* ortholog is present in the budding yeast *S. cerevisiae*, but there is one in the fission yeast *S. pombe* i.e., Clr4 (Nakayama et al., 2001).

To study the function of FmKmt1, the native gene was deleted by homologous integration of a hygromycin resistance cassette into the *F. mangiferae* wild-type strain MRC7560, from now on designated FmWT. Three independent deletion mutants were isolated, Δ*fmkmt1*_T13, T19, and T20, which showed correct integration of the resistance cassette and absence of the wild-type gene as verified by diagnostic PCR (Supplementary Figure 1). All mutants showed an identical phenotype. Hence, Δ*fmkmt1*_T19 was arbitrarily chosen for the complementation approach. Notably, several rounds of transformation failed to obtain complemented strains with a re-integration of the wild-type *FmKMT1* gene at the native locus (*in situ*). Therefore, *FmKMT1* driven by its native promoter and followed by the artificial glucanase terminator from *B. cinerea* (BcTgluc) was transformed ectopically (*ex situ*). This resulted in several positive Δ*fmkmt1*/*FmKMT1*^Ces^ transformants, which showed the presence of *FmKMT1* as determined by diagnostic PCR by the amplification of the genR resistance cassette and the presence of the *FmKMT1* wild-type gene (Supplementary Figure 2B). To verify successful complementation of Δ*fmkmt1* strains, *FmKMT1* expression was analyzed by RT-qPCR. For this, total RNA was extracted from FmWT as well as two independent Δ*fmkmt1* and Δ*fmkmt1*/*FmKMT1*^Ces^ strains grown for 3 days on solid CM. As expected, expression of *FmKMT1* was abolished in Δ*fmkmt1* strains but present in FmWT and Δ*fmkmt1*/*FmKMT1*^Ces^ strains (Figure 1B, Supplementary Figure 2C).

To verify the involvement of FmKmt1 in H3K9 tri-methylation in *F. mangiferae*, FmWT, Δ*fmkmt1* and Δ*fmkmt1*/*FmKMT1*^Ces^ total protein extracts were subjected to western blotting using two different anti-H3K9me3-specific antibodies. H3K9me3 levels were significantly reduced to about 20–50% of FmWT in Δ*fmkmt1* and restored to wild-type level in Δ*fmkmt1*/*FmKMT1*^Ces^ strains (Figure 1C, Supplementary Figure 3A). To determine whether there is an additional H3K9me3-specific SET-domain containing protein present in *F. mangiferae* that may account for the remaining H3K9me3 levels in Δ*fmkmt1*, an additional pBLAST search using the FmKmt1 SET domain as query was performed (Supplementary Figure 4). This analysis revealed seven additional SET-domain containing proteins, including orthologs of FfAsh1, FfSet2, FfSet1, and FfKmt5, which account for H3K36me3 (FfAsh1 and FfSet2), H3K4me1/2/3 (FfSet1) and H4K20me1/2/3 (FfKmt5) in related *Fusarium* species, respectively (Janevska et al., 2018a,b; Bachleitner et al., 2021 minor revision). Next to these, three SET-domain containing proteins with yet unknown functions were detected i.e., FMAN_02077, FMAN_01989, and FMAN_12039, which were excluded from this study, due to their dis-resemblance of published domain structures of Kmt1. Next, we approached an amino acid exchange of K9 for arginine on H3, in order to prevent methylation at this residue. One independent FmH3K9R (T24) mutant was isolated that showed the desired amino acid exchanged (Supplementary Figure 5). Subsequent western blotting was performed as described above using the same anti-H3K9me3-specific antibodies. The signal intensity was comparable to the signal obtained for the Δ*fmkmt1* strains, suggesting that both antibodies are functionally promiscuous or multi-specific at least to some extent in *F. mangiferae* (Figure 1C). It is noteworthy, that FmH3 is 100% (*E*-value 4 × 10^−97^) and 99% (*E*-value 7 × 10^−95^, last amino acid is changed from a serine to an asparagine residue) identical on the protein level to the respective orthologs from *N. crassa* (NCU01635) and *Z. tritici* (HTR2402), respectively, where no unspecific bands were detected in the western blots. To further validate the FmH3K9R strain, H3K9ac levels were determined in the relevant strains. While H3K9ac levels were detectable in FmWT, Δ*fmkmt1*, and Δ*fmkmt1*/*FmKMT1*^Ces^, no signal was obtained for the FmH3K9R strain. Thus, FmKmt1 is a H3K9-specific histone methyltransferase in *F. mangiferae*, and remaining H3K9me3 levels are likely the result of unspecific binding of the used antibodies.

### Lack of FmKmt1 Results in Slightly Reduced Radial Hyphal Growth and Negatively Impacts Conidiation in *F. mangiferae*

**Figure 2:**
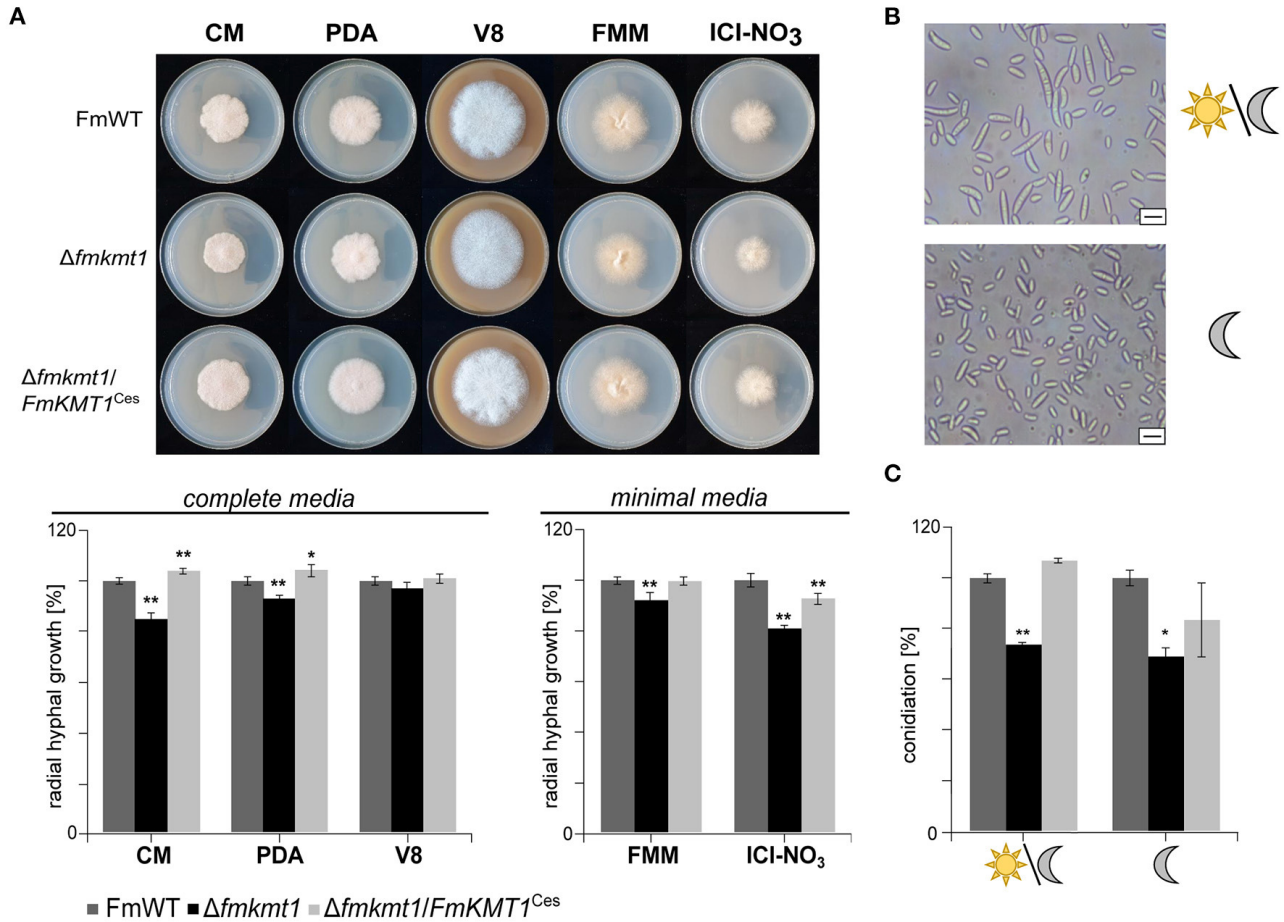
Impact of *FmKMT1* deletion on vegetative growth and asexual development. **(A)** Radial hyphal growth of *F. mangiferae* wild-type (FmWT), Δ*fmkmt1* and Δ*fmkmt1*/*FmKMT1*^Ces^ was assessed on complete media (FCM, PDA, V8) and minimal media (FMM and ICI supplemented with 6 mM NaNO_3_). For this, strains were grown for 5 days at 30°C in the dark. Experiments were performed in biological triplicates. Hyphal growth of FmWT on the respective media was arbitrarily set to 100%. Mean values and standard deviations are shown in the diagram. For statistical analysis a student's *t-*test was performed. Asterisks above the bars denote significant differences in the vegetative growth of the indicated strains compared to the respective wild type, ^*^p < 0.05; ^**^p < 0.001. **(B)** FmWT conidia under L/D and D conditions. FmWT produces under L/D micro- and macroconidia, while under D conditions only microconidia are formed. Bar in the right corner represents 10 μm of size. **(C)** Conidiation assay under L/D and D conditions was performed on vegetable V8 media for FmWT, Δ*fmkmt1*, and Δ*fmkmt1*/*FmKMT1*^Ces^ and assessed after 7 days of incubation. Experiments were performed in triplicates. Conidia production of FmWT was arbitrarily set to 100%. Statistical analysis were performed with a student's *t-*test. Asterisks above the bars denote significant differences in the conidia production of the indicated strains compared to the respective wild type, ^*^p < 0.05; ^**^p < 0.001.

To study the function of FmKmt1 in fungal hyphal growth, FmWT, Δ*fmkmt1*, and Δ*fmkmt1*/*FmKMT1*^Ces^ were grown on different solid minimal and complete media i.e., FMM and ICI as well as V8, PDA, and CM, respectively. Radial hyphal growth was measured after 5 days of growth at 30°C in the dark. In Δ*fmkmt1*, hyphal growth was slightly but significantly reduced on the different media used. More precisely, radial hyphal growth was reduced to about 92 and 80% relative to FmWT, when grown on FMM and ICI, respectively (Figure 2A). Similarly, a slight reduction in hyphal growth was observed for all Δ*fmkmt1* strains, when grown on PDA (93% of FmWT), CM (85% of FmWT), while no growth retardation was detectable for V8. Radial hyphal growth was restored to wild-type levels in Δ*fmkmt1*/*FmKMT1*^Ces^ strains on all tested media (Figure 2A).

*F. mangiferae* is able to form micro- as well as macroconidia (Supplementary Figure 6) (Leslie and Summerell, 2006). While both conidia types appear upon cultivation of FmWT under 18 h/6 h light-dark conditions, only microconidia were generated when cultivated in constant darkness (Figure 2B, Supplementary Figure 6). To study the involvement of FmKmt1 in asexual reproduction, FmWT, Δ*fmkmt1* and Δ*fmkmt1*/*FmKMT1*^Ces^ were grown for 7 days at 20°C and either under 18 h/6 h light-dark conditions or alternatively in constant darkness. Subsequent quantification of conidia showed a significantly reduced amount under both conditions. Conidia production was reduced to 74 and 69% when grown under 18 h/6 h light-dark and constant darkness, respectively, in Δ*fmkmt1* and conidia production was rescued to wild-type levels in Δ*fmkmt1*/*FmKMT1*^Ces^ (Figure 2C). Thus, FmKmt1 slightly though significantly affects fungal growth and conidiation in *F. mangiferae*.

### FmKmt1 Is Largely Dispensable for Wild Type-Like Stress Response Toward Osmotic Stressors

In *F. verticillioides* and *Zymoseptoria tritici* the homologs of FmKmt1 i.e., FvDim5 and ZtKmt1, respectively are involved in osmotic stress response (Gu et al., 2017; Möller et al., 2019). To explore the involvement of FmKmt1 and H3K9me3 in fungal stress response also in *F. mangiferae*, FmWT, Δ*fmkmt1*, and Δ*fmkmt1*/*FmKMT1*^Ces^ strains were grown in the presence of osmotic stressors, i.e., CM supplemented with either 1 M NaCl or 1 M sorbitol. Fungal growth on non-supplemented CM served as control. Radial hyphal growth was slightly but significantly elevated to 110 and 113% in the presence of 1 M NaCl and 1 M sorbitol, respectively, in Δ*fmkmt1* compared to FmWT and rescued to wild-type levels in Δ*fmkmt1*/*FmKMT1*^Ces^ (Supplementary Figure 7), suggesting that FmKmt1 only slightly but significantly contributes to the osmotic stress response in *F. mangiferae*.

### Deletion of *FmKMT1* Increases Beauvericin Biosynthesis in *F. mangiferae*

*F. mangiferae* harbors 52 putative SM biosynthetic gene clusters (SMBGC). However, so far only 18 can be assigned to their respective products as determined by experimental and/or bioinformatics analyses (Supplementary Table 2; Niehaus et al., 2016a). Among these are gibberellic acid (Bömke and Tudzynski, 2009), fusaric acid (Niehaus et al., 2014b; Studt et al., 2016a), gibepyrone D (Janevska et al., 2016) and the two red pigments bikaverin (Wiemann et al., 2009), and fusarubins (Studt et al., 2012). Next to this, *F. mangiferae* harbors the SMBGC recently shown to be involved in the production of the mycotoxin beauvericin (Niehaus et al., 2016b). All of these SMs are readily quantifiable by HPLC-MS (Niehaus et al., 2017). Up to now, only the production of the two red pigments bikaverin and fusarubins under nitrogen-limiting conditions, as well as for fusaric acid under nitrogen-sufficient conditions has been verified yet (Niehaus et al., 2016a).

To analyze the impact of FmKmt1 on the above-mentioned SMs in *F. mangiferae*, their production levels were assessed in FmWT and three independent Δ*fmkmt1* strains. For this, strains were grown in liquid ICI medium supplemented with different nitrogen sources as described previously (Niehaus et al., 2016a). In the case of *F. mangiferae*, all analyzed SMs except gibberellins and gibepyrone D were identified in at least one of the chosen conditions (Table 1).

**Table 1:** SM production in *F. mangiferae* under inducing conditions.

**Metabolite**	**60 mM glutamine**	**6 mM glutamine**	**6 mM NaNO_**3**_**
Gibberellins	-	-	-
Bikaverin	-	+	-
Fusarubins	-	+	+
Fusaric acid	+	-	-
Beauvericin	+	+	+
Gibepyrone D	-	-	-

-*The metabolite was not present/not detected in respective media*.

+*Presence of metabolite in respective media*.

Deletion of *FmKMT1* did not reveal significant alterations for most of the analyzed SMs, except for beauvericin. Beauvericin biosynthesis in low nitrogen conditions (6 mM NaNO_3_) was affected upon loss of FmKmt1 resulting in an 13–26-fold increase in beauvericin production in Δ*fmkmt1* strains compared to FmWT, while beauvericin biosynthesis remained unaffected in the other culture conditions. Biosynthesis of fusaric acid, bikaverin, and fusarubins was not affected by *FmKMT1* deletion under any of the analyzed conditions (Supplementary Figure 8). To summarize, biosynthesis of the pigments bikaverin and fusarubins as well as the mycotoxins fusaric acid and beauvericin by FmWT is stimulated through the here applied culture conditions. Lack of FmKmt1 has no impact on the biosynthesis of the pigments and fusaric acid but affects beauvericin biosynthesis under defined conditions.

### FmKmt1 and Wild-Type H3K9me3 Levels Are Crucial for Fusapyrone Biosynthesis in *F. mangiferae*

**Figure 3:**
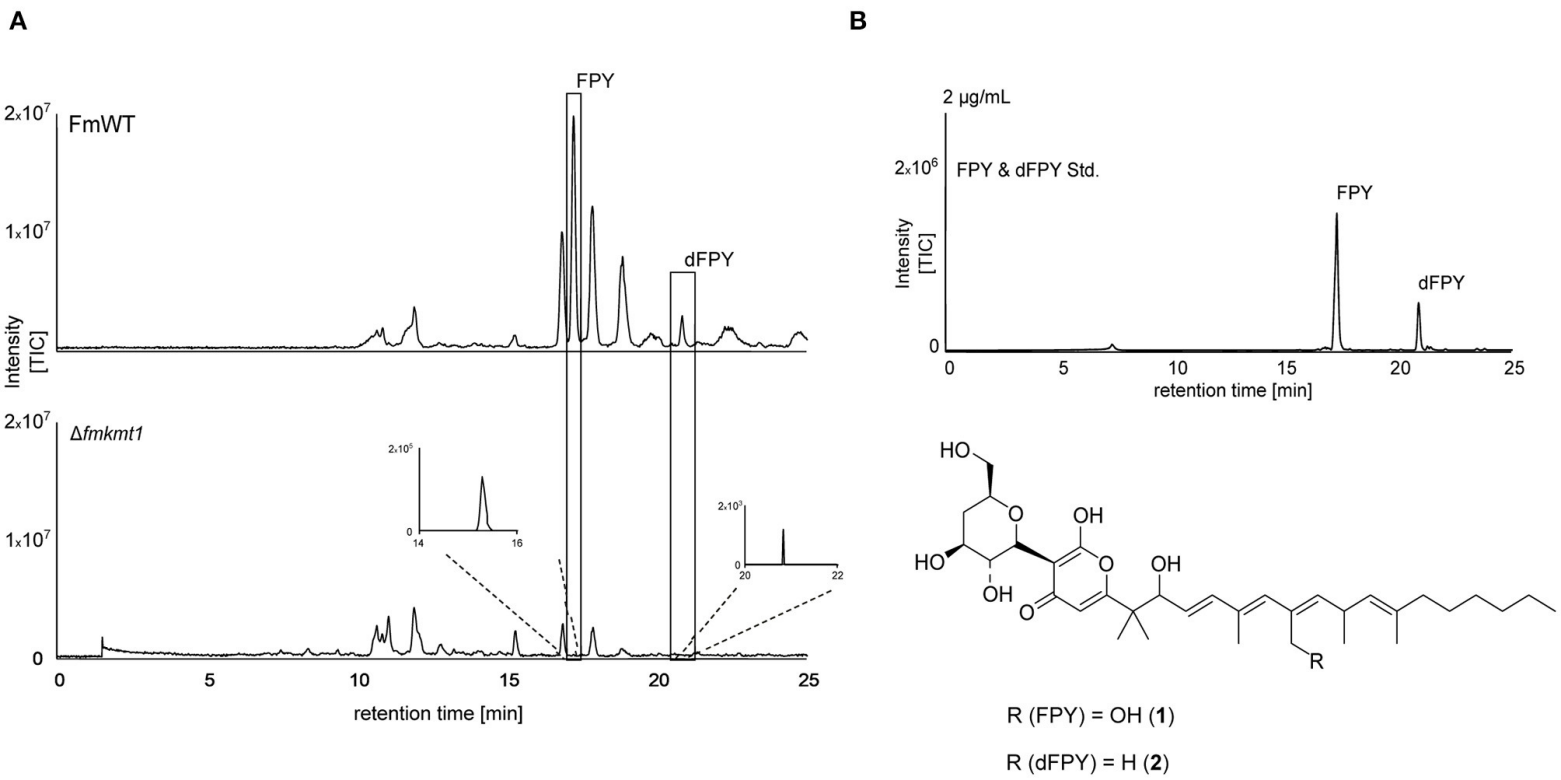
Fusapyrone (FPY) and deoxyfusapyrone (dFPY) chemical profiles in *F. mangiferae*. **(A)** HPLC-HRMS chromatograms of FPY (1) and dFPY (2) production in the *FmKMT1* deletion strain (Δ*fmkmt1*) compared to the wild-type strain (FmWT). For this, FmWT and three independent Δ*fmkmt1* strains were cultivated in FPY-inducing media for 7 days at 30°C. Experiments were performed in triplicates. The measurement of fungal supernatants show that only traces of FPY and dFPY are produced by Δ*fmkmt1* compared to FmWT. **(B)** HPLC-HRMS chromatogram of FPY and dFPY standards (applied in a concentration of 2 μg/mL). Structures of the γ-pyrones FPY and dFPY are shown below. TIC chromatograms (positive ESI-mode) range from *m/z* 100 to 1,000.

When comparing HPLC-MS/MS profiles, a prominent peak was detected upon growth of the different strains in liquid ICI medium supplemented with 6 mM NaNO_3_ (FPY-inducing culture condition) in the *F. mangiferae* wild-type strain that was almost absent from the Δ*fmkmt1* strain (Figure 3A). The peak could not be assigned to any of the known products from FFSC members. Exact mass determination suggested *m*/*z* 607.3826 [M+H]^+^, which corresponds to the mass of fusapyrone (FPY, calculated *m*/*z* 607.3846 for [C_34_H_54_O_9_+H]^+^). Another less prominent peak present in FmWT but nearly missing from Δ*fmkmt1* extracts gave an exact mass of *m*/*z* 591.3882 [M+H]^+^, which corresponds to deoxyfusapyrone (dFPY, calculated *m*/*z* 591.3897 for [C_34_H_54_O_8_+H]^+^). Comparing retention times and exact masses of the newly identified peaks with the respective standard compounds verified the presence of both, FPY and dFPY in FmWT (Figure 3B). Both compounds were indeed almost completely abolished in Δ*fmkmt1* (Figure 3A), suggesting that FmKmt1 is crucial for wild type-like FPY biosynthesis in this fungus. To verify this hypothesis, FmWT, Δ*fmkmt1*, and Δ*fmkmt1*/*FmKMT1*^Ces^ were cultivated under FPY-inducing conditions, and production levels were quantified. Biosynthesis of FPY and dFPY was reduced to about 10% of FmWT in Δ*fmkmt1* and nearly restored to wild-type level in Δ*fmkmt1*/*FmKMT1*^Ces^ (Supplementary Figure 9). To evaluate whether these finding are connected with H3K9me3, the FmH3K9R strain was cultivated under FPY-inducing conditions together with FmWT and Δ*fmkmt1*. Subsequent SM analysis showed that similar to Δ*fmkmt1*, FPY, and dFPY levels were abolished in FmH3K9R, while FPY and dFPY were produced in sufficient amounts for FmWT (Supplementary Figure 10).

### Fusapyrone Biosynthesis Is Nitrogen Dependent but Not Determined by the pH

**Figure 4:**
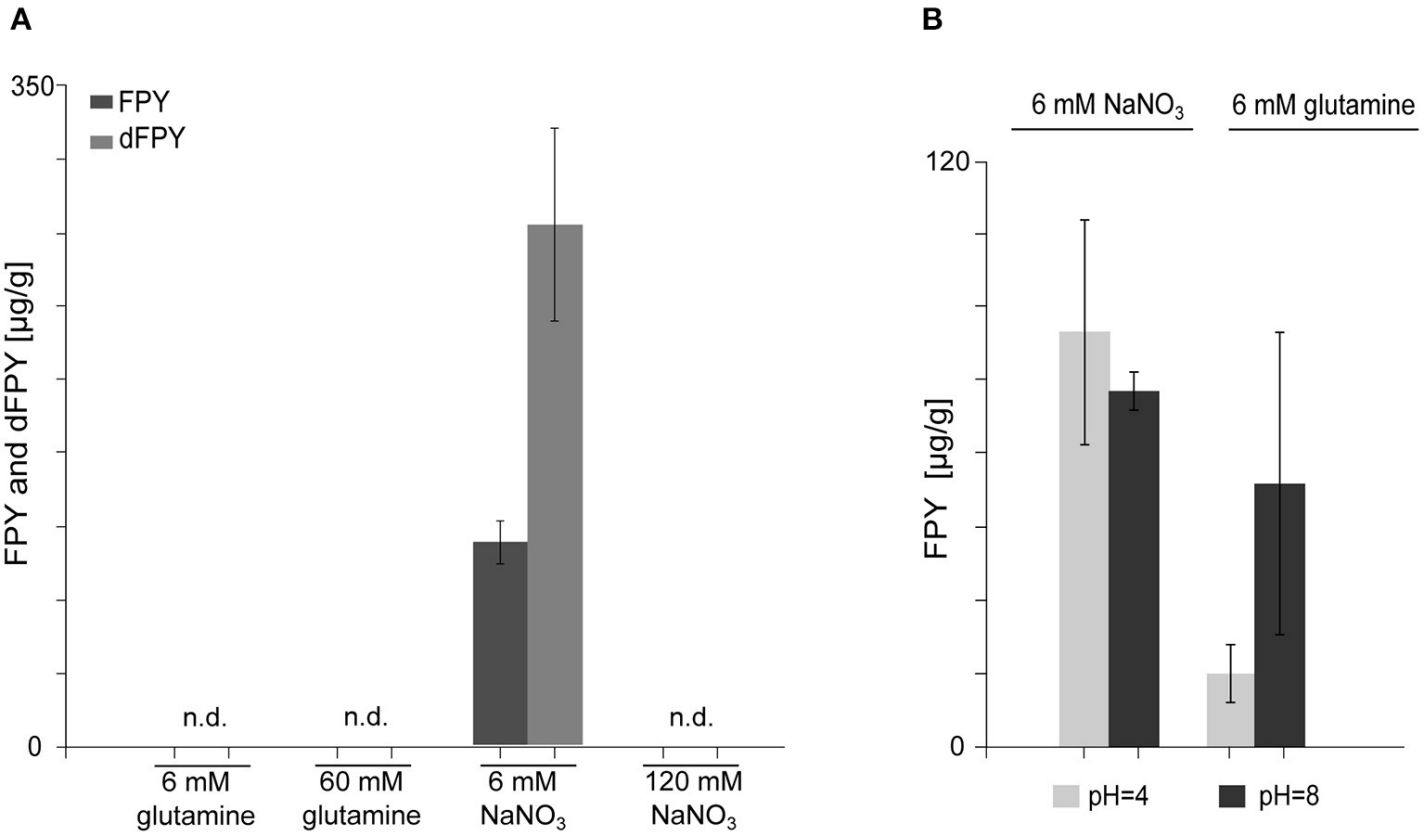
Biosynthesis of fusapyrone (FPY) and deoxyfusapyrone (dFPY) is N-repressed but independent of the ambient pH in *F. mangiferae*. **(A)** Measurement of FPY and dFPY levels with HPLC-HRMS. Production of FPY by FmWT under standard laboratory culture conditions i.e., liquid ICI supplemented with 6 mM or 60 mM glutamine and 6 mM or 120 mM NaNO_3_ for 7 days at 30°C. FPY and dFPY are only produced in detectable amounts by FmWT in media supplemented with 6 mM NaNO_3_. Experiments were performed in triplicates. Mean values and standard deviations are shown in diagram. n.d., not detected in the supernatant. **(B)** Quantitative determination of FPY production levels by FmWT measured with HPLC-HRMS. FmWT was cultivated in 6 mM NaNO_3_ and 6 mM glutamine for 7 days at 30°C in the dark. The pH was set prior inoculation to respective levels using 100 mM NaH_2_PO_4_ (pH 4) and 100 mM Na_2_HPO_4_ (pH 8). pH levels were controlled after 7 days using pH paper. Experiments were performed in triplicates. Mean values and standard deviations are shown in diagram.

Nitrogen availability and source greatly influences SM biosynthesis under laboratory conditions, in the related *Fusarium* species *F. fujikuroi* (Wiemann et al., 2013). To analyze whether FPY biosynthesis is nitrogen dependent, FmWT was cultivated in liquid ICI supplemented with either 6 or 60 mM glutamine and 6 or 120 mM NaNO_3_, respectively_._ Subsequent SM analysis verified that FPY biosynthesis is nitrogen-repressed as neither FPY nor dFPY were detectable upon cultivation in 120 mM NaNO_3_ and 60 mM glutamine (FPY-repressing conditions). Cultivation in 6 mM glutamine led to amounts below the quantitation level; while levels of FPY and dFPY were high in media supplemented with 6 mM NaNO_3_ (Figure 4A).

As described earlier *F. fujikuroi*-inoculated medium supplemented with 6 mM glutamine differs from medium supplemented with 6 mM NaNO_3_ with regard to the pH (Studt et al., 2012). While the pH is more acidic upon cultivation in 6 mM glutamine, cultivation in NaNO_3_ drives media conditions more alkaline, which is also true for *F. mangiferae*. To test whether FPY biosynthesis is favored in basal over acidic pH conditions, FmWT was cultivated in liquid ICI supplemented with either 6 mM NaNO_3_ or 6 mM glutamine under the addition of 100 mM NaH_2_PO4 for acidic conditions (pH 4) or 100 mM Na_2_HPO4 (pH 8) for a more alkaline media environment (Peñalva et al., 2008). Subsequently, SM analysis showed that although alkaline pH resulted in slightly elevated FPY biosynthesis when grown in ICI supplemented with 6 mM glutamine, no differences were observed for the different pH conditions when grown in ICI supplemented with 6 mM NaNO_3_. Both FPY and dFPY accumulated in either media condition to nearly the same extent (Figure 4B). This suggest that NaNO_3_ induces FPY biosynthesis independent of the pH. In summary, the regulation of FPY biosynthesis appears to be strictly repressed by nitrogen availability and independent of pH.

### FmPKS40 Is Involved in Fusapyrone Biosynthesis in *F. mangiferae*

**Figure 5:**
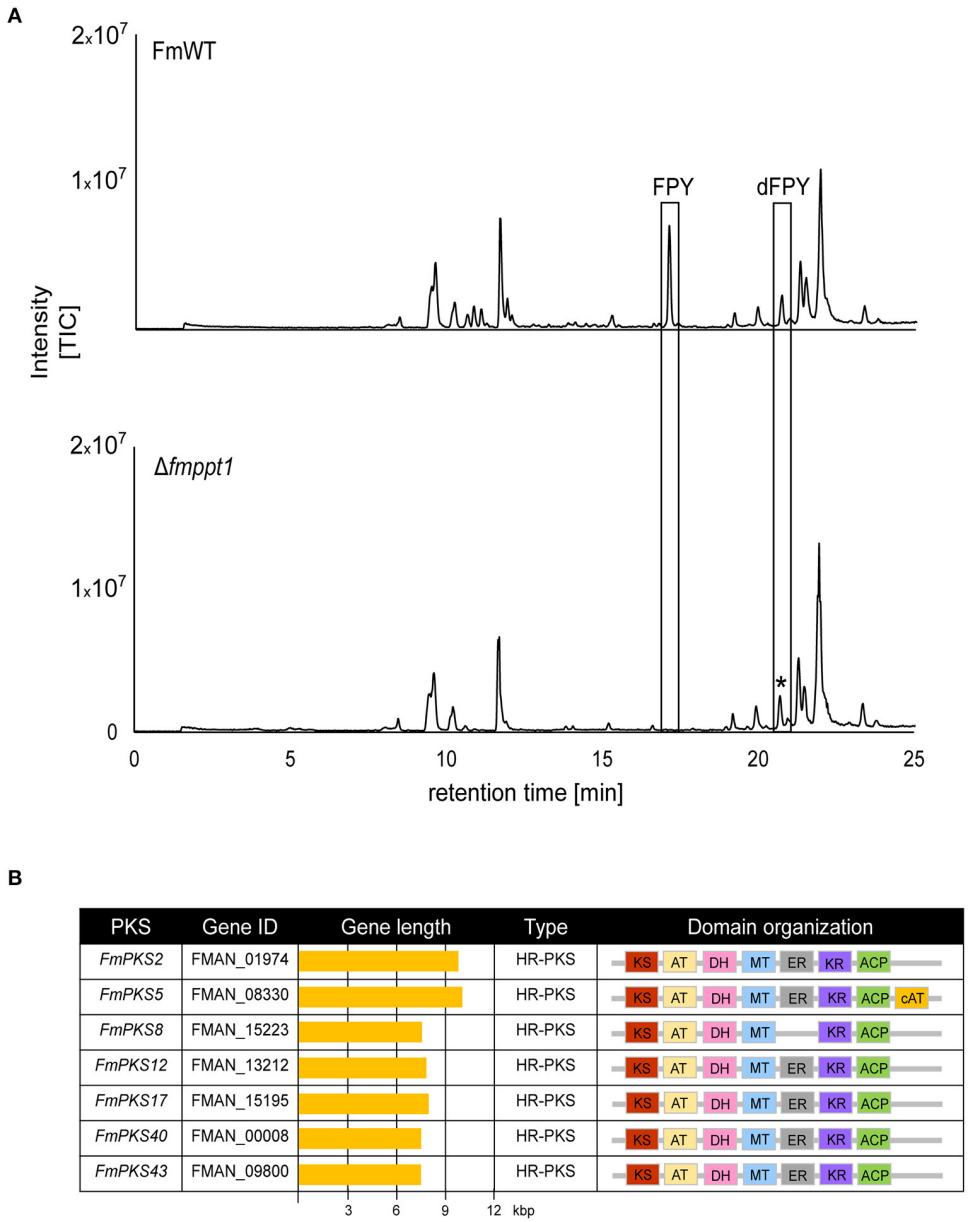
Fusapyrone (FPY) and deoxyfusapyrone (dFPY) are PKS-derived metabolites. **(A)** HPLC-HRMS chromatograms of supernatants from *F. mangiferae* (FmWT) and Δ*fmppt1* grown for 7 days at 30°C in the dark in liquid ICI supplemented with 6 mM NaNO_3_ as sole nitrogen source. Experiments were performed in triplicates. Peaks for FPY and dFPY (boxed) are present in FmWT but absent from Δ*fmppt1* cultures. The asterisk (^*^) indicates that the observed signal in the Δ*fmppt1* chromatogram with a similar retention time as dFPY is distinct from dFPY since the signal has a different mass. TIC chromatograms (positive ESI-mode) range from *m/z* 100 to 1,000. **(B)** Overview of PKS-encoding candidate genes for FPY biosynthesis. Sequences were retrieved from the publicly available genome sequence of *F. mangiferae* MRC7560 (Niehaus et al., 2016a). Depicted are the key enzymes, gene IDs, gene lengths, PKS types as well as the predicted domain organizations. Domain organization was analyzed using the NCBI Conserved Domain (Marchler-Bauer et al., 2017), InterPro (Blum et al., 2021), SBSPKSv2 (Khater et al., 2017); and the PKS/NRPS Analysis Web-site (Bachmann and Ravel, 2009). KS (red), keto synthase; AT (yellow), acyltransferase; DH (pink), dehydratase; MT (blue) C-methyltransferase; ER (gray) enoylreductase; KR (violet), ketoreductase; ACP (green), acyl carrier protein; cAT (orange), carnitine acyltransferase.

The chemical structure suggests that FPY and dFPY are derived from the condensation of 11 acetyl-CoA subunits resulting in the formation of a hexa-methylated undecaketide with a γ-pyrone ring system (Hiramatsu et al., 2006). These chemical features support the idea of a PKS origin for both substances. To further support this assumption, we deleted the putative 4′-phosphopantetheinyl transferase (Ppt) encoded by *PPT1* in *F. mangiferae*. Functionality of most PKSs (and non-ribosomal peptide synthetases, NRPSs) depends on post-translationally modified acyl carrier protein (ACP) domains by 4′-phosphopantetheinyl transferases (PPTases) (Neville et al., 2005; Horbach et al., 2009; Zainudin et al., 2015; Derbyshire et al., 2019). The putative Ppt homolog in *F. mangiferae*, FmPpt1, was identified by determining the ortholog using QuartetS (Yu et al., 2011). The predicted protein, FMAN_09282 (FmPpt1) is the putative ortholog of the *F. fujikuroi* Ppt1 (FFUJ_06276) (Wiemann et al., 2012). The respective gene, *FmPPT1*, was deleted by homologous integration of a hygromycin resistance cassette into FmWT. Four independent deletion mutants were obtained, Δ*fmppt1*_T6, T10, T25, and T27 that showed correct integration of the resistance cassette and absence of the wild-type gene as verified by diagnostic PCR (Supplementary Figures 11A,B). Strains lacking Ppt1 are lysine auxotroph, a phenotype that may be explained by the missing 4′-phosphopantetheinylation of the apo α-aminoadipate reductase involved in lysine biosynthesis (Ehmann et al., 1999). Thus, successful generation of Δ*fmppt1* mutants was additionally verified by absence of growth on solid ICI media without lysine (Supplementary Figure 11C). Subsequent cultivation of FmWT and Δ*fmppt1* strains under FPY-inducing conditions provided further evidence for a PKS origin of FPY and dFPY as both compounds were present in the FmWT but missing from Δ*fmppt1* samples (Figure 5A).

Minimal PKSs contain an acyltransferase (AT) domain involved in the selection of α-carboxyacyl extender units, a ketoacyl-synthase (KS) domain that performs the decarboxylative condensation of the extender unit to the growing polyketide chain and an ACP covalently shuttling polyketide intermediates between the domains. Highly reducing (HR)-PKSs differ from non-reducing (NR)-PKSs in that they possess additional domains that perform programmed modifications at the α or β carbon. These typically include a ketoreductase (KR), a dehydratase (DH), and an enoylreductase (ER), that result in the formation of a β-hydroxyl group, an α/β-double bond, or a β-methylene. Next to this, the incorporation of C-methyl groups is typically derived from C-methyltransferase (CMet) domains that function in the transfer of methyl groups from S-adenosylmethionine (SAM) to β-ketoacyl-ACP substrates (Chooi and Tang, 2012; Storm et al., 2018). Notably, fungal PKSs function iteratively and modification of the resulting enzyme-bound poly-β-keto intermediate is governed by the activity of the KR, DH, and/or ER domains after each condensation event (Cox, 2007). FPY shows typical features of a reduced polyketide exemplified by the partially reduced carbon chain containing six C-methylated α-carbons. Bioinformatic analysis of HR-PKSs with intrinsic CMet domains but yet uncharacterized functions gave seven candidate genes (Figure 5B). Noteworthy, FmPKS8 does not harbor an ER domain required for the formation of ß-methylene units present in FPY. However, FMAN_15222 directly downstream of FmPKS8 and transcribed from a bi-directional promoter encodes an ER domain-containing protein that may act in *trans* on the growing polyketide chain. *Trans*-acting enoyl reductases have been identified also in other fungi exemplified by LovC or ApdC present in the lovastatin and aspyridone gene clusters (Kennedy et al., 1999; Wasil et al., 2013). Thus, FmPKS8 was included in the following analyses.

**Figure 6:**
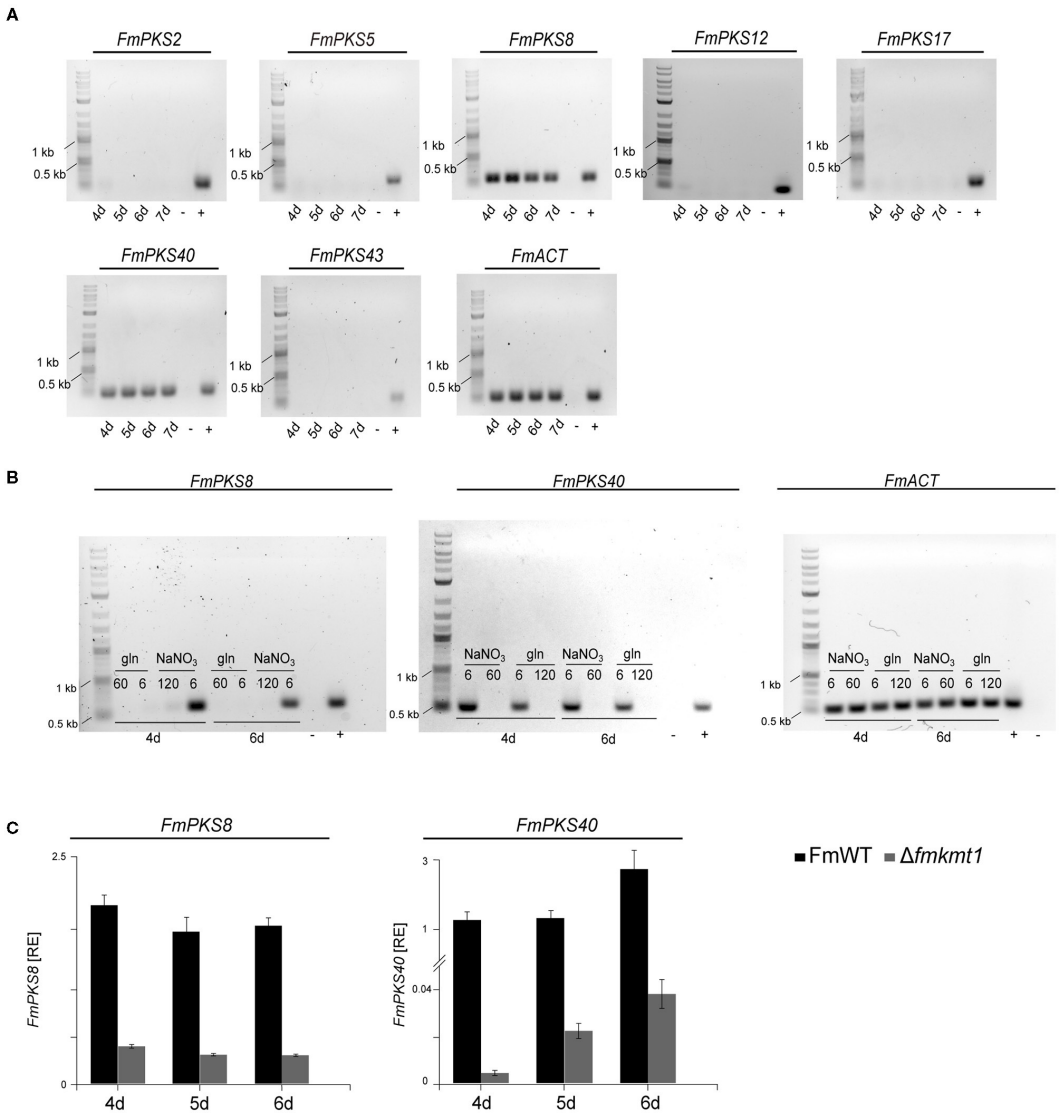
Expressional analysis of fusapyrone (FPY) and deoxyfusapyrone (dFPY) candidate genes in *F. mangiferae*. **(A)** Semi-quantitative PCR of candidate PKS genes for FPY and dFPY production. *F. mangiferae* (FmWT) was cultivated for 4–7 days in FPY-inducing media and subsequently RNA was extracted from lyophilized mycelia und transcribed to cDNA. Primer used for the detection of gene expression are listed in Supplementary Table 1. **(B)** Semi-quantitative PCR of gene expression in liquid ICI supplemented with different N-sources. FmWT was cultivated for 4 and 6 days in FPY-inducing [additionally 6 mM glutamine (gln)] and FPY-repressing conditions. Extracted RNA was then transcribed to cDNA for semi-quantitative PCR reaction. Primer pairs used for *FmPKS8* and *FmPKS40* expressional analysis are listed in Supplementary Table 1. **(C)** For the comparison of transcription levels, FmWT and Δ*fmkmt1* were cultivated for 4–6 days in FPY inducing conditions. From lyophilized mycelium RNA was extracted and cDNA synthesized. Expression levels of *FmPKS8* and *FmPKS40* were measured *via* RT*-*qPCR. Mean values and standard deviations are shown. RE, relative normalized expression. For cDNA control actin was amplified using the primers Actin_F//R. As size marker GeneRuler 1 kb Plus DNA Ladder (NEB) was used. As positive control (+) FmWT gDNA was used, while as negative control (-) sterile water was used.

To determine the expression of the identified PKS-encoding genes, FmWT was cultivated under FPY-inducing conditions. As FPY and dFPY were detectable 7 but not 3 days post inoculation (Supplementary Figure 12), mycelium for subsequent expression analysis was harvested 4, 5, 6, and 7 days post inoculation. Here, only *FmPKS8* and *FmPKS40* showed expression under FPY-inducing conditions (Figure 6A). Next, FmWT was cultivated for 4 and 6 d under FPY-inducing as well as under FPY-repressing conditions. Subsequently, expression profiles were determined for both *FmPKS8* and *FmPKS40*. In line with the chemical analyses, the respective transcripts were low (*FmPKS40*) or absent (*FmPKS8*) for 6 mM glutamine as well as under non-favorable conditions as determined by semi-quantitative PCR (Figure 6B). Next, FmWT and Δ*fmkmt1* were cultivated under FPY-inducing conditions, and *FmPKS8* as well as *FmPKS40* expression was quantified by RT-qPCR after 4, 5, and 6 days of growth. Again, expression profiles of both *FmPKS8* and *FmPKS40* followed production levels, i.e., high in FmWT and significantly reduced in Δ*fmkmt1* to 20 or 1.5% of FmWT values, respectively (Figure 6C).

**Figure 7:**
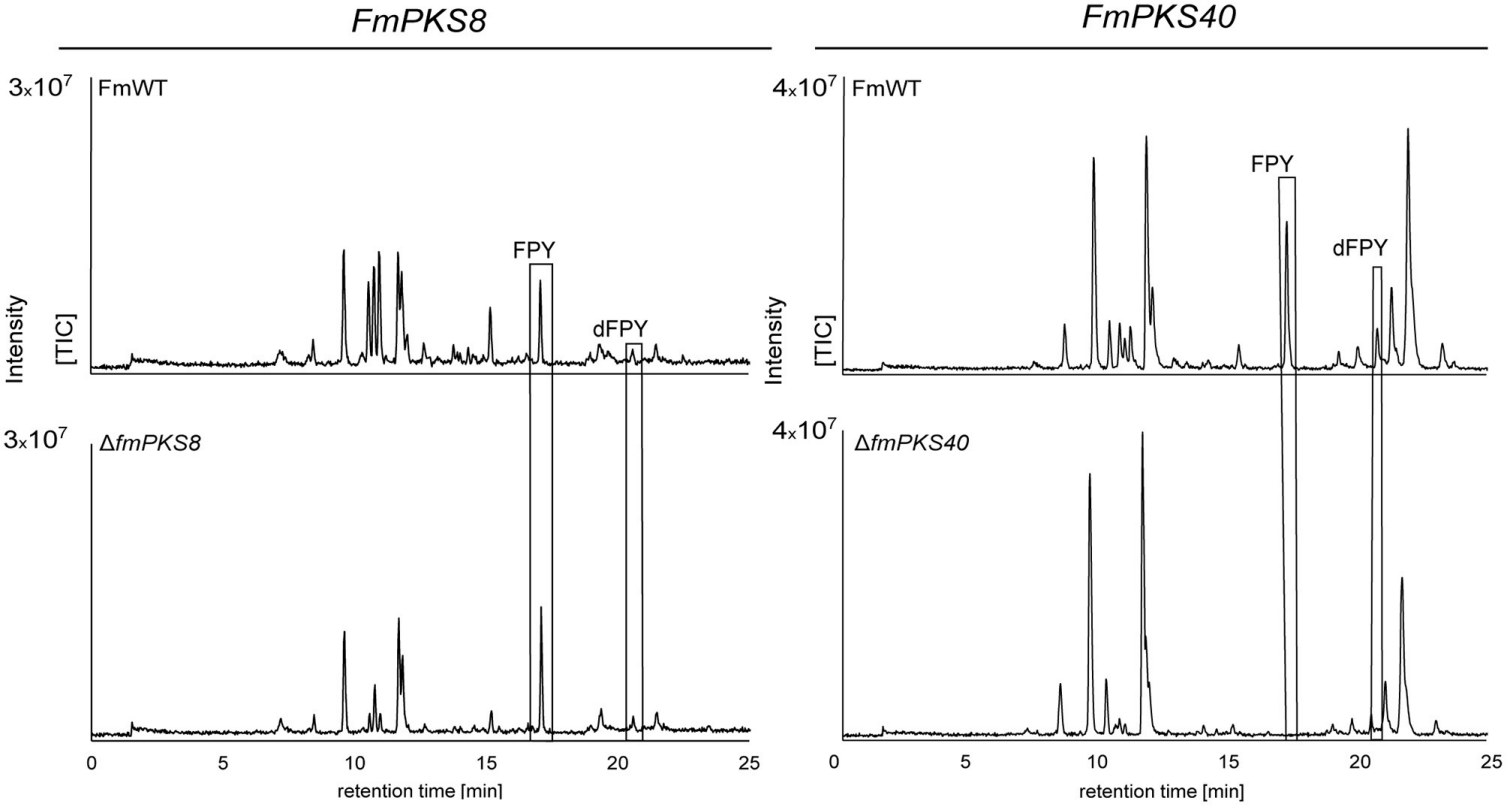
HPLC-HRMS chromatograms of fusapyrone (FPY) and deoxyfusapyrone (dFPY) production in Δ*fmPKS8* and Δ*fmPKS40* cultures. *F. mangiferae* wild-type (FmWT) and indicated deletion strains were cultivated for 7 days in FPY-inducing media. Experiments were performed in triplicates. The measurement of fungal supernatants revealed that no FPY and dFPY is produced in Δ*fmPKS40* cultures, while Δ*fmPKS8* retained wild type-like production levels. TIC chromatograms (positive ESI-mode) range from *m*/*z* 100 to 1,000.

To provide further evidence, that either *FmPKS*8 (FMAN_15223) or *FmPKS40* (FMAN_00008) is responsible for FPY biosynthesis, partial gene deletions of *FmPKS8* and *FmPKS40* was performed as attempts to delete the complete open reading frame were unsuccessful. Therefore, deletion cassettes were generated designated to replace the KS domain of *FmPKS8* and *FmPKS40* (~2 kb; see Supplementary Figures 13A, 14A), that is strictly required for polyketide biosynthesis (Sabatini et al., 2018). Partial gene deletion in the case of *FmPKS8* and *FmPKS40* yielded three and two independent transformants, Δ*fmPKS8* _T9, T21 and T49, as well as Δ*fmPKS40*_ T26 and T27, respectively (Supplementary Figures 13B, 14B). Next, SM analysis in liquid ICI under FPY-inducing conditions revealed that, while Δ*fmPKS8* strains retained wild type-like FPY and dFPY production levels, FPY and dFPY biosynthesis was completely abolished in Δ*fmPKS40* strains (Figure 7). Therefore, FmPKS40 is responsible for FPY biosynthesis in *F. mangiferae*.

### Fusapyrone Biosynthesis Is Facilitated by a Seven-Gene Cluster

**Figure 8:**
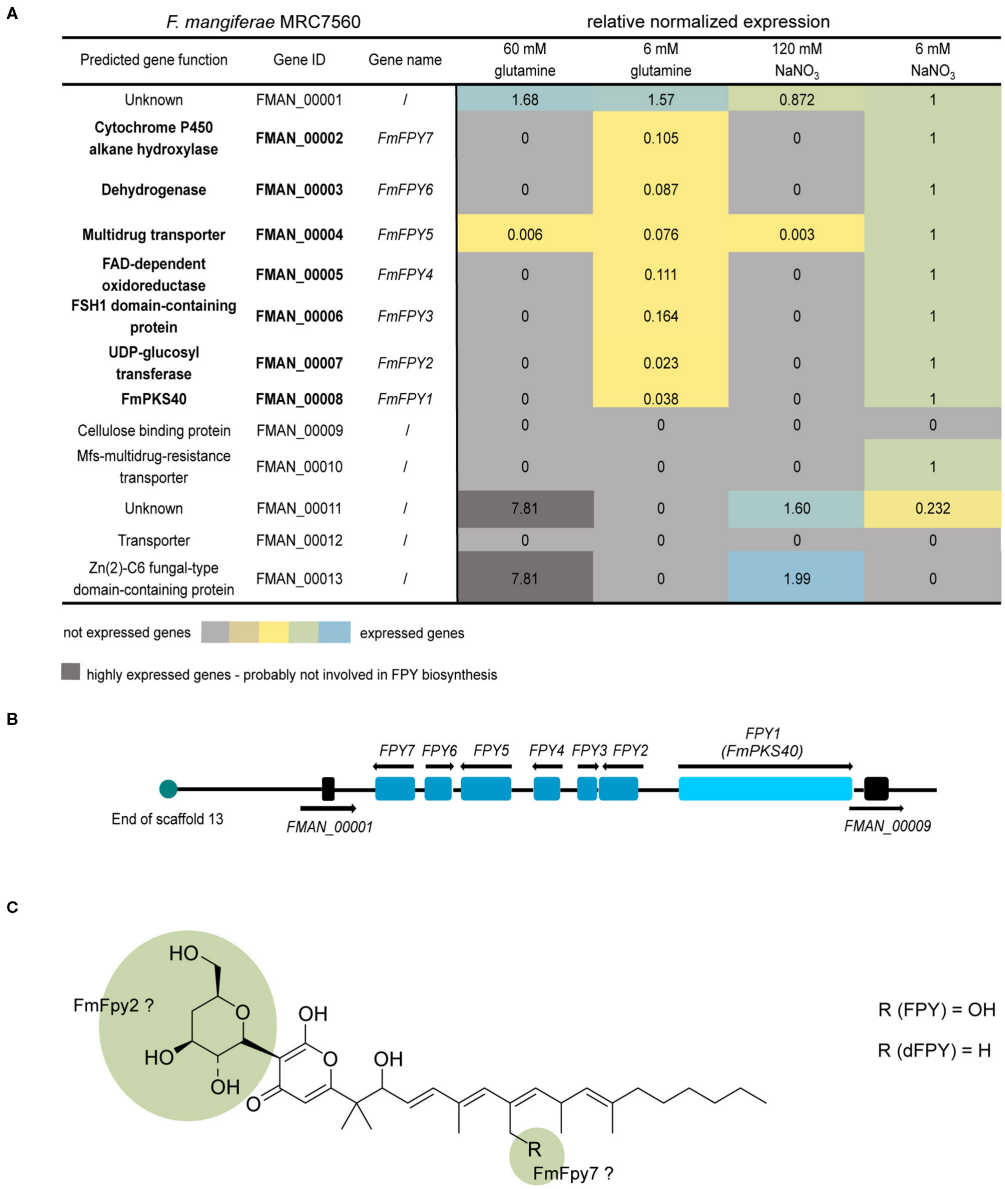
Co-expression studies of genes putatively involved in fusapyrone (FPY) and deoxyfusapyrone (dFPY) biosynthesis. **(A)** Heat map of FmFPY SMBGC co-expression studies. Here FmWT was cultivated at 30°C for 4 days under different N-sources and concentrations (ICI + 60/6 mM glutamine or 120/6 mM NaNO_3_). Subsequently RNA was extracted and transcribed into cDNA for RT-qPCR analysis. For data normalization, the house -keeping genes actin, GPD and β-tubulin were used. Primers are listed in Supplementary Table 1. **(B)** FmFPY1 is indicated as light blue rectangle, while associated cluster genes FmFPY2 - FmFPY7 are indicated as blue rectangles. The upstream border gene FMAN_00001 and the downstream border gene FMAN_00009 are depicted in black rectangles. Black arrows show translation direction. Turquoise circle indicates end of *F. mangiferae* scaffold 13. **(C)** Structure formula of FPY and dFPY. Green highlighted parts of the structure formula indicate functions of tailoring enzymes on the molecule.

Next to the key enzyme-encoding gene, SMBGCs often contain additional genes encoding tailoring enzymes, transporter- or pathway-specific transcription factors (Hang et al., 2016). Genes in the proximity of *FmPKS40* include, among others, genes with predicted functions as an UDP-glycosyltransferase (FMAN_00007), a cytochrome P450 hydroxylase (FMAN_00002), a fungal-type transcription factor (FMAN_00013), or transporters (FMAN_00004, FMAN_00010, FMAN_00012) (Figure 8A). To experimentally determine the FPY cluster borders, the FmWT was cultivated in FPY-inducing (additionally in 6 mM glutamine) and FPY-repressing conditions. Fungal cultures were harvested 4 days post inoculation. Expression of genes adjacent to *FmPKS40* in different media was analyzed using RT-qPCR (Figure 8A). Next to *FmPKS40*, six additional genes located upstream of *FmPKS40* i.e., FMAN_00007-FMAN_00002, were expressed under FPY-inducing conditions, while transcripts were absent from FPY-repressing conditions (Figure 8A). The gene FMAN_00009 immediately downstream of *FmPKS40* was not expressed under any of the here investigated conditions, while FMAN_00001 transcripts were present under any of the tested conditions and not affected by high nitrogen. Thus, FMAN_00001 as well as FMAN_00009 likely do not belong to the FPY gene cluster. To sum up, the putative FmPKS40 gene cluster involved in FPY biosynthesis likely consists of seven genes (FMAN_00008-FMAN_00002) from now on designated *FmFPY1* through *FmFPY7* (Figure 8B). Where applicable putative functions of proteins encoded by the cluster genes are highlighted in Figure 8C.

### Fusapyrone Biosynthesis in Other Members of the FFSC

**Figure 9:**
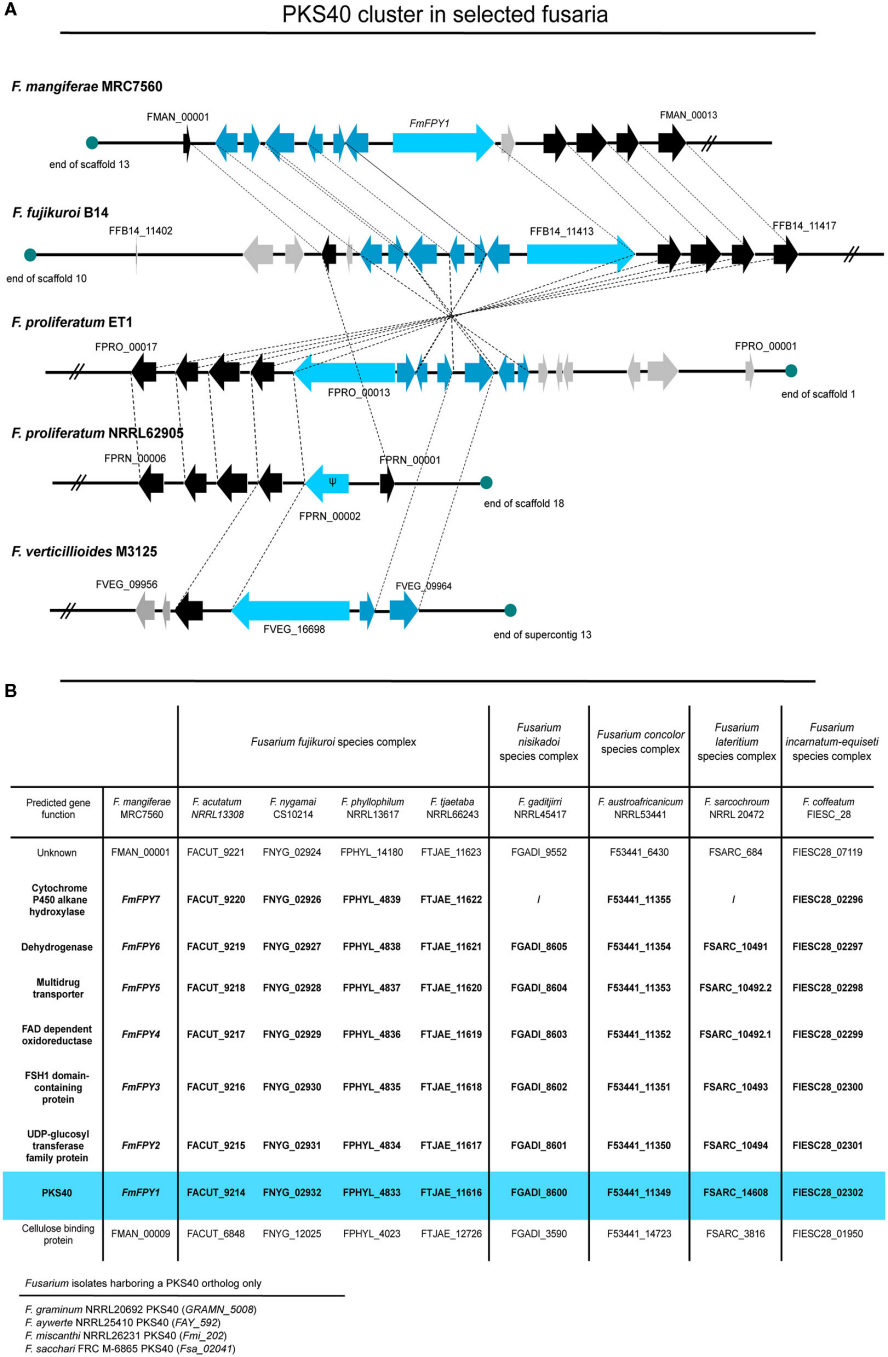
Comparison of PKS40 cluster in different members of the FFSC. **(A)** The putative PKS40 gene cluster is only present in *F. mangiferae* MRC7560*, F. fujikuroi* B14, and *F. proliferatum* ET1. For *F. proliferatum* NRRL62905 only a remnant of PKS40 and no other putative cluster genes are present, while for *F. verticillioides* M3125 an orthologous PKS40 gene and only two cluster genes can be found. Light blue arrows indicate the respective PKS40 key enzyme, while blue arrows are designated putative cluster genes. Black arrows represent genes that are orthologes in other species and grey arrows show genes which do not have closely related orthologes. Ψ designates a pseudogene. **(B)** Table of several other *Fusarium* species from the FFSC and other *Fusarium* species complexes with orthologes PKS40 gene clusters. Data was retrieved from NCBI and an extended pBLAST search was performed of FmFPY1-FmFPY7 against other fusaria.

Using QuartetS (Yu et al., 2011), we identified putative FmPKS40 homologs also in other members of the FFSC, including *F. verticillioides* M3125 (*Fv*M3125), *F. proliferatum* NRRL62905 (*Fp*NRRL62905), ET1 (*Fp*ET1), and *F. fujikuroi* B14 (*Ff* B14), all of which were readily available for further analyses. Comparison of the genomic regions of the FPY SMBGC present in all four fusaria indicates that only *Ff* B14 and *Fp*ET1 harbor the entire FPY gene cluster as determined in *F. mangiferae*, while the cluster appears abrogated in *Fp*NRRL62905 and *Fv*M3125 (Figure 9A). Furthermore, an extended pBLAST search against FmPKS40 was performed to identify putative orthologs of the FmFPY cluster in other *Fusarium* isolates from the FFSC but also from other *Fusarium* species complexes (Figure 9B). Here, *FmFPY1-FmFPY7* were detected in several other *Fusarium* species including members from the *Fusarium nisikadoi*-, *Fusarium concolor*-, *Fusarium lateritium*-, and *Fusarium incarnatum-equiseti* species complexes. For some other isolates an orthologous *FmPKS40* gene was detected, however, the putative cluster genes could not be detected *via* pBLAST search. Interestingly, for *F. gaditjirri* and *F. sarcochroum* no ortholog of *FmFPY7* is present, suggesting that the cluster is abrogated after *FmFPY6* in these two species.

**Figure 10:**
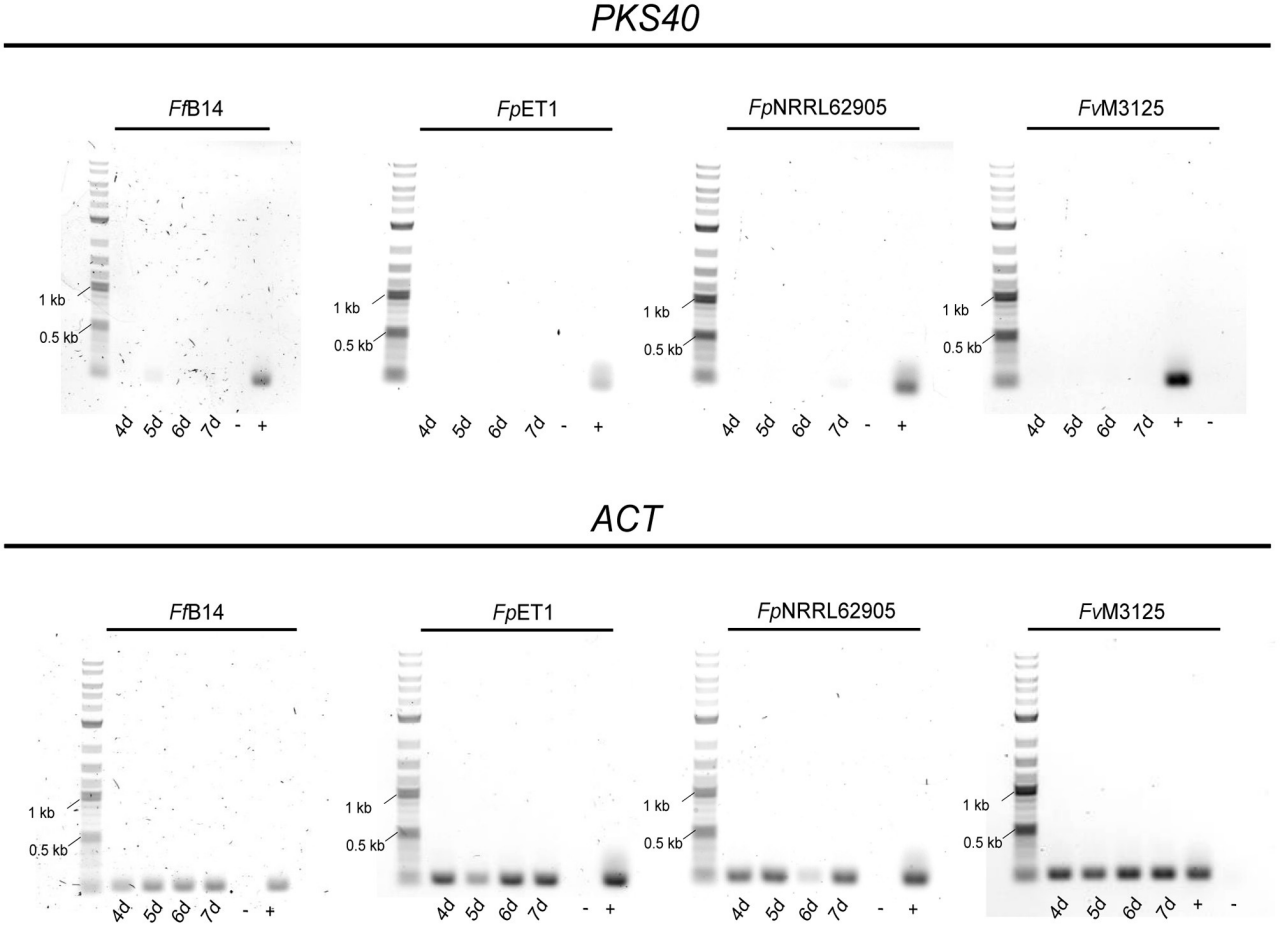
Semi-quantitative gene expression analysis of *PKS40* in other members of the FFSC. The respective *Fusarium* strains were cultivated for 4–7 days in FPY-inducing conditions. Subsequently, RNA extraction and cDNA synthesis were performed. Gene expression was tested with the primer pair FfPKS40_F//FmPKS40_R for the indicated *Fusarium* species. As cDNA control the housekeeping gene actin was amplified using the primers Actin_F//R. As size marker the GeneRuler 1 kb Plus DNA Ladder (NEB) was used. As positive control (+) respective *Fusarium* sp. gDNA was used, while as negative control (-) sterile water was used.

To determine whether *FPY* gene regulation is conserved among the different fusaria, the wild-type strains *Fv*M3125, *Fp*NRRL62905, *Fp*ET1, and *Ff* B14 were cultivated under FPY-inducing conditions as determined for *F. mangiferae*, and cultures were harvested 4–7 dpi. Subsequent semi-quantitative PCR showed very weak expression for *FfFPY1*, while no *FPY1* expression was detectable for *F. verticillioides* as well as for both *F. proliferatum* strains (Figure 10). To analyze whether FPY is synthesized in small amounts in *Ff* B14, the fungus was cultivated for 7 days in FPY-inducing conditions. Subsequent SM analysis revealed that no FPY was produced by *Ff* B14 albeit weak expression of its respective *FfPKS40* orthologous gene. Thus, next to *F. mangiferae* only *Fp*ET1 and *Ff* B14 harbor the entire *FPY* gene cluster as determined here. However, while for *F. mangiferae* liquid ICI supplemented with 6 mM NaNO_3_ is favoring FPY biosynthesis, for the other two species neither *FPY* gene expression nor FPY biosynthesis was detectable under the conditions used here. This suggests that other environmental stimuli are required for FPY biosynthesis in these species.

## Discussion

Here, we identified and characterized FmKmt1, the functional ortholog of the H3K9me3-specific methyltransferase NcDIM-5 in *N. crassa* (Tamaru and Selker, 2001). FmKmt1 is involved in the establishment of H3K9me3 also in *F. mangiferae*. Lack of FmKmt1 in *F. mangiferae* has only minor influence on fungal development and the production of most known SMs with the exception of beauvericin that was highly induced in the mutant under certain conditions. Next to this, FmKmt1 has also stimulatory roles for the production of other SMs, exemplified here by the discovery of FPY, a γ-pyrone previously only known from *F. semitectum* (Evidente et al., 1994; Hiramatsu et al., 2006). By a combination of bioinformatics and reverse genetics, here we link FPY biosynthesis with the responsible PKS (FmFpy1), delineate the cluster borders by co-expression studies and provide insights into its regulation in *F. mangiferae*.

### FmKmt1 Only Slightly Affects Fungal Development and Osmotic Stress Resistance in *F. mangiferae*

FmKmt1 is largely dispensable for wild type-like vegetative growth in *F. mangiferae* with an observed radial growth reduction of about 10–15% on complete and minimal media, respectively. These findings are in line with studies on *A. fumigatus, F. verticillioides, B. cinerea*, or *M. oryzae* (Palmer et al., 2008; Pham et al., 2015; Zhang et al., 2016; Gu et al., 2017), in which deletion of the respective homologs, *AfclrD, FvDIM5, BcDIM5*, and *MoKMT1*, respectively, also had only a slight impact on vegetative growth. No growth defect was detectable for *A. nidulans clrD*Δ on solid as well as in liquid media (Reyes-Dominguez et al., 2010). Notably, clrD in *A. nidulans* is ~200 amino acids longer compared to FmKmt1 in *F. mangiferae*. However, no known domain is predicted within the extended N-terminus. While FmKmt1 homologs appear to have only little impact on radial hyphal growth in the aforementioned Ascomycota, a more drastic impact is described for *N. crassa, E. festucae*, and *Z. tritici*. Strains deleted for the respective homologous genes, *NcDIM-5, EfclrD*, and *ZtKMT1*, respectively, showed severe growth retardations (Tamaru and Selker, 2001; Freitag et al., 2004; Chujo and Scott, 2014; Möller et al., 2019). Furthermore, RNAi-mediated gene silencing of *LmDIM5* in the plant pathogen *Leptosphaeria maculans* resulted in an average reduction of radial hyphal growth of about 35% (Soyer et al., 2014).

Osmoadaption is an important indicator of fungal fitness to cope with environmental fluctuations. Therefore, fungal stress tests on solid media supplemented with 1 M sorbitol and 1 M NaCl were performed. Fungal hyphal growth was only slightly but significantly increased in the presence of osmotic stressors (sorbitol and NaCl) in Δ*fmkmt1* compared to the wild type. Similarly, Δ*fvdim5* strains showed increased hyphal growth on media supplemented with osmotic stressors in *F. verticillioides* (Gu et al., 2017). Here, western blot analysis of *FvDIM5* deletion strains showed highly elevated levels of the mitogen-activated protein kinase FvHog1. Hog1 is strongly associated with osmotic stress response in the budding yeast *S. cerevisiae* (Brewster et al., 1993; Westfall et al., 2004). On the contrary, osmotic stress applied to *Z. tritici* Δ*ztkmt1* revealed hypersensitivity toward both sorbitol and NaCl (Möller et al., 2019). However, studies on the influence of H3K9me3 for osmoadaption in diverse fungal species will be necessary to conclude about its importance.

FmKmt1 influences asexual development in *F. mangiferae*, as conidiation was reduced to about 70% of the wild type. These results are in agreement with *F. verticillioides, A. fumigatus*, and *B. cinerea* as lack of the *FmKMT1* homologs *FvDIM5, AfclrD*, and *BcDIM5* resulted in declined conidiation (Palmer et al., 2008; Zhang et al., 2016; Gu et al., 2017). Contrary to this, asexual development remained unaffected upon deletion of *MoKMT1* and *clrD* in *M. oryzae* and *A. nidulans*, respectively (Reyes-Dominguez et al., 2010; Pham et al., 2015). Thus, H3K9me3 appears to be more relevant for some, e.g., *Z. tritici, L. maculans, N. crassa*, or *E. festucae*, than for other fungal species, e.g., *A. nidulans, B. cinerea, M. oryzae*, or *Fusarium* spp.

### Lack of H3K9me3 Has Distinct Effects on Secondary Metabolism in *F. mangiferae*

Altered H3K9me3 levels have shown to affect secondary metabolism in several filamentous fungi. For *F. verticillioides* deletion of *FvDIM5* resulted in elevated gene expression of two melanin biosynthesis-related genes. Furthermore, increased production of the mycotoxin fumonisin B_1_ was observed for this species (Gu et al., 2017). Nearly abolished levels of H3K9me3 in *E. festucae* revealed the de-repression of genes involved in the biosynthesis of the mycotoxins lolitrem and ergot alkaloids. In the wild type, these SMs are solely expressed upon symbiotic interactions *in planta*. However, deletion of *EfclrD* resulted in the production of both virulence factors in axenic culture (Chujo and Scott, 2014). For *A. nidulans*, lack of clrD lead to an increased gene expression of sterigmatocystin cluster genes *aflR* and *stcO*, concomitant with a slight increase in sterigmatocystin biosynthesis (Reyes-Dominguez et al., 2010). For *L. maculans* RNA-seq data of *LmDIM5* knockdown strains suggests that LmDim5 influences SM-associated genes (Soyer et al., 2014).

*F. mangiferae* harbors 52 SM key enzyme-encoding genes (Niehaus et al., 2016a), of which thus far only 18 can be linked to the corresponding product. However, not much is known regarding their production under standard laboratory conditions in this species. FmKmt1 was found to be largely dispensable for their production in *F. mangiferae*. Next to bikaverin, fusarubins, and fusaric acid that were known to be produced (Niehaus et al., 2016a), we show here that *F. mangiferae* produces the depsipeptide beauvericin in all analyzed culture conditions. Lack of FmKmt1 even increases beauvericin biosynthesis in 6 mM NaNO_3_. In *F. fujikuroi* IMI58289, beauvericin biosynthesis is silenced by H3K27me3 and only synthesized in sufficient amounts after H3K27 de-methylation (Niehaus et al., 2016b). The effect of H3K27me3 in beauvericin biosynthesis in *F. mangiferae* remains elusive at this point. However, it is noteworthy that upon deletion of the Kmt1 homologs NcDIM-5 and ZtKmt1, H3K27me3 re-locates to genomic region formerly enriched for H3K9me3 (Jamieson et al., 2016; Möller et al., 2019). Whether this is also true for *F. mangiferae* remains to be determined. Next to known SMs, a prominent but so far cryptic peak was identified in the wild-type extracts but nearly missing in Δ*fmkmt1*, which was identified as the γ-pyrone FPY in the course of this study. Almost complete absence of FPY from Δ*fmkmt1* strains indicate that wild type-like H3K9me3 levels are necessary for FPY biosynthesis in this species. These findings are supported by the absence of FPY in culture extracts of FmH3K9R mutant. Next to the genes involved in FPY biosynthesis, *FmPKS8* expression was also high in the wild-type strain and downregulated upon deletion of *FmKMT1*. This suggest that at least another yet unknown SM is affected similarly by loss of FmKmt1. Unfortunately, the promiscuity of both anti-H3K9me3-specific antibodies used in our study preclude genome-wide ChIP-sequencing in *F. mangiferae* at this point. Thus, we cannot determine whether SM genes are directly targeted by FmKmt1. A negative correlation of H3K9me3 levels and SM gene expression was thus far only shown for *E. festucae* and *A. nidulans* by gene-specific ChIP-PCR (Reyes-Dominguez et al., 2010; Chujo and Scott, 2014). However, for the latter, recent genome-wide ChIP-sequencing studies revealed that SMBGCs in *A. nidulans* are largely free of this histone mark (Gacek-Matthews et al., 2016), therefore, rather pointing toward a regulation in *trans*. Moreover, recent combined ChIP-sequencing and transcriptome studies in *Z. tritici* revealed that H3K9me3 is not involved in the regulation of SMBGCs (Möller et al., 2019), despite the relatively high abundance of H3K9me3 (Schotanus et al., 2015). Thus, the involvement of H3K9me3 in SM gene regulation needs to be carefully addressed in the future.

### A Seven-Gene Cluster Facilitates Fusapyrone Biosynthesis

Polyketides represent an important class of compounds that are extremely diverse in structure and function. In contrast to microbial PKSs, fungal PKSs function iteratively i.e., utilizing a single set of elongation and domains modifying the α or ß carbon of the growing polyketide chain through every round of elongation to synthesize a precisely modified polyketide. The structure of the resulting polyketide is thus determined by the presence and defined use of domains within the PKS. Furthermore, tailoring enzymes located within close proximity of the key enzyme may further modify the polyketide backbone resulting in a huge diversity of PKS-based products. FPY is an undecaketide and thus likely synthesized from acetyl-CoA functioning as starter unit and the addition of 10 malonyl-CoA extender units by successive Claisen-condensations. Next to this, FPY shares some rare features: C-glycosylated 4-deoxyglucose at C-3, a *gem*-dimethyl group at C-13, and an α-ß to ß-⋎ double bond shift at C-20.

The *gem*-dimethyl group in polyketides are relatively rare; most examples are from bacterial polyketides, including the potential anticancer agents epothilone, pederin, bryostatin, disorazol, and the siderophore yersiniabactin (Miller et al., 2002; Hang et al., 2017; Meinke et al., 2018). The features that control whether zero, one, or two methyl groups are transferred to a substrate are largely unknown. The first example on fungal polyketides harboring a *gem*-dimethyl group was shown for *Trichoderma virens* (Hang et al., 2017). Here, the CMet domain of Tv6-931, a HR-PKS of *T. virens*, is involved in mono- and di-methylation of a produced tetraketide that is mono-methylated at the first and second α-carbon and di-methylated at the third α-carbon. Notably, Hang et al. (2017) proposed that a carnitine acyltransferase (cAT) domain present in Tv6-931 is involved in releasing and recapturing the PKS-derived product thereby facilitating complete *gem*-dimethylation by the CMet. However, *gem*-dimethylation also happened in absence of the cAT domain. During FPY biosynthesis mono-C-methyl groups are transferred to the tetra-, penta-, hexa- and heptaketide, while two C-methyl groups are transferred to the nonaketide, suggesting that the CMet domain is programmed to selectively catalyze two successive C-α-methylation reactions of the nonaketide, while other α-carbons are non- or mono-methylated only (Chan et al., 2009; Hang et al., 2017). No cAT domain was detected in FmFpy1, suggesting that the *gem*-dimethyl group transfer to the nonaketide is independent of this domain in FPY biosynthesis.

The α-ß to ß-⋎ double bond shift at C-20 is another rare feature in FPY biosynthesis. The formation of *cis*-double bonds has so far only been studied in bacterial-derived SMs. Unlike fungal PKS, bacterial PKS are structured in modules, which can be used also in iterative fashion (Chen and Du, 2016). In general, *cis*-to-*trans* isomerization is proposed to be determined by the stereochemical outcome of the KR domain by the production of either 3R-alcohols (*trans*) or 3S-alcohols (*cis*). Subsequently the DH domain recognizes the substrate and dictates the distinct double bond formation (Caffrey, 2003; Keatinge-Clay, 2007). This mechanism has been studied for epothilones and tylosins (Tang et al., 2004; Keatinge-Clay, 2007). However, some exceptions are known e.g., for biosynthesis of hypothemycin or the anti-angiogenic substance borrelidin (Vergnolle et al., 2011). For the latter, the shift from *trans*-to-*cis* is not determined by KR-dependent stereochemistry, but rather the DH accepts also 3R-alcohols for the *cis*-double bond formation (Vergnolle et al., 2011). However, the involvement of another protein, not located in the borreledin SMBGC was not excluded. In the case of hypothemycin *cis*-double bond formation at C-5 is achieved by a glutathione S-transferase (Reeves et al., 2008). Nothing is known for fungal-derived SMs thus far.

Next to this, the posttranslational modification of microbial SMs with deoxysugars is unusual but exemplifies the plethora of natural product modification (Lombó et al., 2009). So far, the deoxygenation pathways for C-2, C-3, and C-6 are fully elucidated (Hallis and Hung-Wen, 1999), while only little is known for C-4 deoxygenation. The only example is known from the soil bacterium *Streptomyces venezuelae*. Here, a hexose sugar is modified *via* a two-step sequential deoxygenation process including an amino sugar intermediate yielding in a 3-(dimethylamino)-3,4,6-trideoxyhexose moiety (desosamine), which then is covalently attached to the final products (Zhao et al., 2001; Szu et al., 2005). However, a similar mechanism is unlikely in the case of FPY biosynthesis, as *F. mangiferae* lacks the essential genes for a similar reaction, at least within the FPY gene cluster. While the origin of the 4′-deoxyglucose moiety remains opaque at this point, its transfer to C-3 is most likely mediated by the C-glycosyltransferase encoded by FmFpy2 (Figure 8C). C-glycosylation is a scarce mechanism in natural product modification and not described for fungal-derived SMs yet. Still, some C-glycosyltransferases were identified in plants, bacteria and mammals (Putkaradze et al., 2021). Generally, C-glycosyltransferases catalyze the formation of a β-glycosidic bond between a carbon atom of an aromatic ring to a previously activated sugar moiety (Putkaradze et al., 2021), as observed in the case of FPY. Next to this, the hydroxyl group present at C-33 and discriminating between FPY and dFPY, is likely to be installed by FmFpy7 encoding a cytochrome P450 (Cyp450) often involved in hydroxylation of fungal SMs, such as in *F. fujikuroi* apicidin F, fusarubin, or fusarin biosynthesis (Figure 8C) (Studt et al., 2012; Niehaus et al., 2013, 2014a). However, it is interestingly to note that for *F. gaditjirri* and *F. sarcochroum* no *FmFPY7* ortholog encoding a Cyp450 gene is located in close proximity to the respective PKS40 gene cluster (Figure 9B), which hints toward a different final product in these fusaria. No putative function can be predicted for the remaining genes (FmFpy3-FmFpy6). FmFpy3 and FmFpy6 encode a FSH1 domain-containing protein with a predicted serine hydrolase domain and a dehydrogenase with predicted oxidoreductase function, respectively. Notably, a FSH serine hydrolase (*FUS5*) is found in the fusarin gene cluster in *F. fujikuroi*, which is not essential for its biosynthesis, albeit co-expressed (Niehaus et al., 2013). Similar findings were made for a dehydrogenase gene (*FSR4*) in the fusarubin gene cluster where deletion of the respective gene did not influence the metabolite production (Studt et al., 2012). Notably, an α/β- hydrolase fold domain is predicted for FmFpy3. The same domain structure is present in LovG from *Aspergillus terreus*, which is indispensable for lovastatin biosynthesis (Xu et al., 2013). FmFpy5 encodes a major facilitator superfamily (MFS) transporter often encoded within SMBGCs, though less is known about its function. For example, in *F. fujikuroi* the MFS transporter encoded in the FUB gene cluster is responsible for the export of the respective product, fusaric acid, by means of detoxification (Studt et al., 2016a). Similarly, for *F. graminearum* and *Fusarium sporotrichioides* the MFS transporter FgTri12 is indispensable for a targeted export of trichothecene mycotoxins, hence self-protection in both species (Alexander et al., 1999; Menke et al., 2012). On the contrary, deletion of *APF11*, encoding a MFS transporter within the *APF* gene cluster involved in apicidin F biosynthesis in *F. fujikuroi*, did not affect apicidin F production (Niehaus et al., 2014a). Future studies on the FPY biosynthesis are required to shed more light on this.

To summarize, FmKmt1 is a H3K9me3-specific methyltransferase in *F. mangiferae*. Lack of FmKmt1 only slightly though significantly impacted fungal hyphal growth, osmotic stress response and asexual development. With the exception of beauvericin, most of the known SMs from *F. mangiferae* remained unaffected upon deletion of *FmKMT1*, but FmKmt1 was found to influence the expression of *FmPKS8* and *FmPKS40*. The latter was identified to be involved in FPY biosynthesis, a polyketide that was previously not known to be produced by members of the FFSC. FPY biosynthesis was repressed by nitrogen but independent of the ambient pH. By a reverse genetics approach, we identified the key enzyme (FmPKS40) involved in FPY biosynthesis and through co-expression studies we elucidated the *FPY* gene cluster in *F. mangiferae*.

## Data Availability Statement

The original contributions presented in the study are included in the article/Supplementary Material, further inquiries can be directed to the corresponding author/s.

## Author Contributions

LS, AA-K, JS, and H-UH contributed to conception and design of the study. AA-K, FL, and SK performed the experiments. AA-K, FL, SK, JS, H-UH, and LS were involved in data analysis. AA-K, FL, and LS wrote the manuscript. All authors contributed to manuscript revision, read, and approved the submitted version.

## Conflict of Interest

The authors declare that the research was conducted in the absence of any commercial or financial relationships that could be construed as a potential conflict of interest.
