# Supplementary material for: Biosynthesis of Fusapyrone Depends on the H3K9 Methyltransferase, FmKmt1, in Fusarium mangiferae
Source: Front Fungal Biol. 2021 Jul 6;2:671796. doi: 10.3389/ffunb.2021.671796 (PMC10512364; doi:10.3389/ffunb.2021.671796)
Supplement: Supplementary file 1 [file Data_Sheet_1.pdf]

# *Supplementary Material*

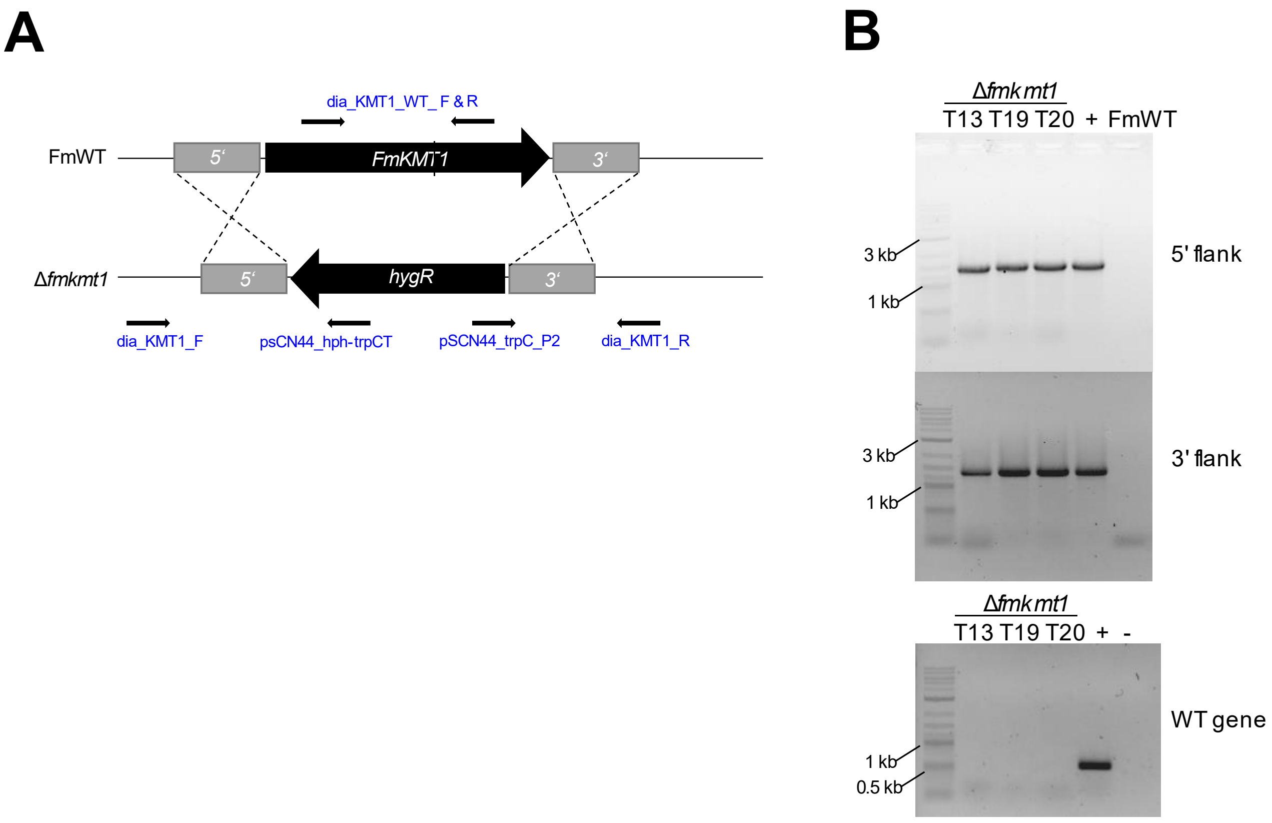


**Figure S1: Strategy and verification of Δ*fmkmt1* deletion mutants by diagnostic PCR. (A)** Deletion and verification strategy of Δ*fmkmt1* mutants. Primer pairs for diagnostic PCRs are depicted in blue. **(B)** Verification of deletion *via* homologous recombination with the hygR cassette. Homologous integration was verified by the presence of the upstream region (5' flank) with the primers Dia_FmKMT1_F//pCSN44_hph-trpC-T and the downstream region (3' flank) by Dia_FmKMT1_R//pCSN44_trpC_P2. Absence of wild-type gene was checked with Dia_WTKMT1_F//R for three independent transformants Δ*fmkmt1* T13, T19 and T20. For 5' and 3' Δ*fmkmt1* gDNA prior single spore isolation was used as positive control (+), while FmWT gDNA served as + control for wild-type (WT) gene diagnostic PCR. As size marker 1 kb Plus DNA ladder (NEB) was used.

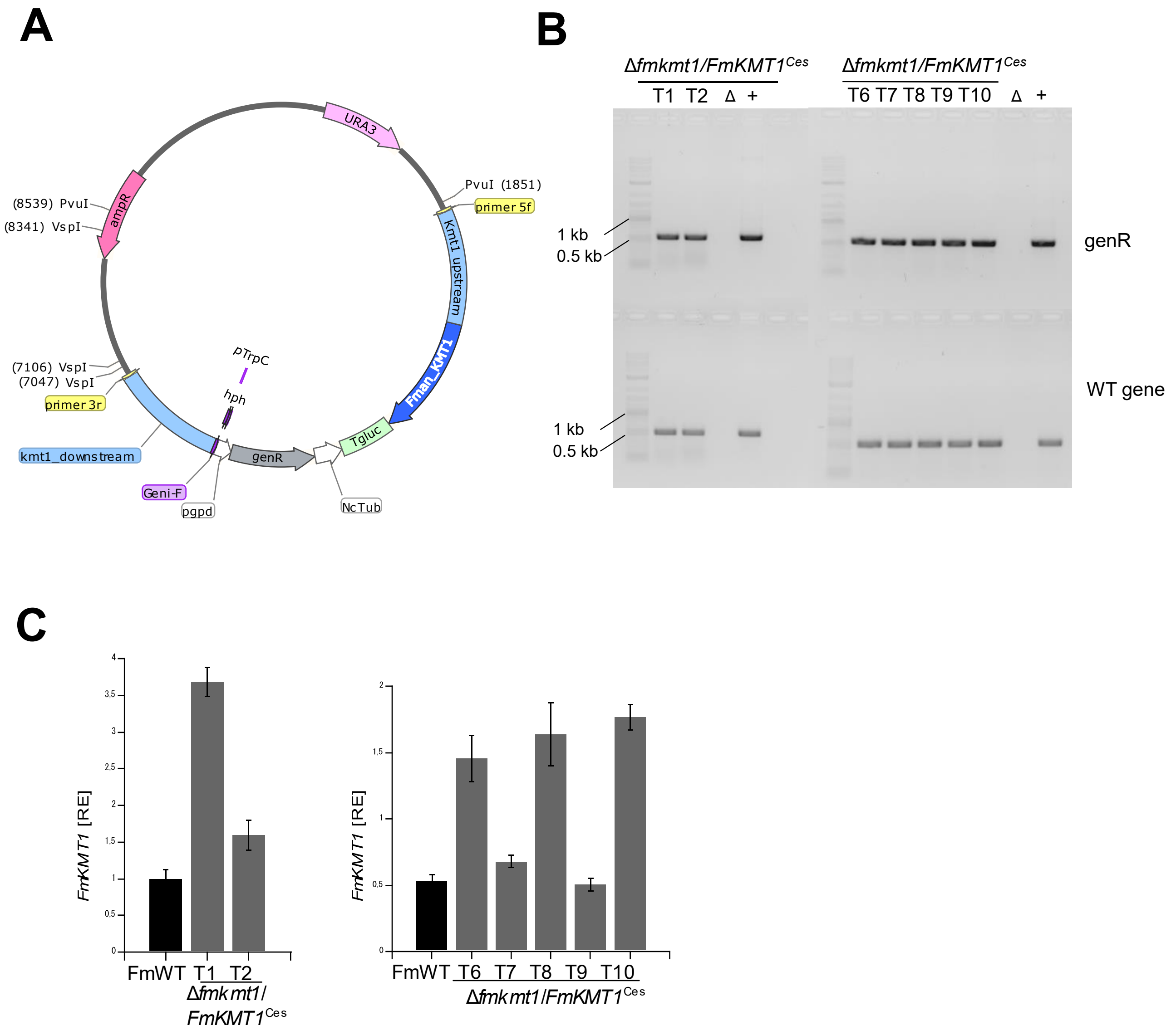


**Figure S2: Complementation strategy and verification of Δ*fmkmt1*/*FmKMT1*$^{Ces}$ by diagnostic PCR. (A)** Strategy for complementation of Δ*fmkmt1*_T19. Enzymes used for restriction are shown. **(B)** Verification of gained transformants. Complementation was achieved by *ex-situ* integration of the native *FmKMT1* wild-type gene of *F. mangiferae* MRC7560 (FmWT) into Δ*fmkmt1* T19. Genomic DNA was isolated from overall seven geneticin-resistant transformants and correct re-integration of the native *FmKMT1* was verified by diagnostic PCR. For this, the amplification of the genR cassette (GeniTglucR/GeniF) together with the wild-type *FmKMT1* gene was performed using the primer pair Dia_WTKMT1F//R. As negative control gDNA of the recipient Δ*fmkmt1* T19 strain was used, while as positive control (+) for geneticin resistance the pΔ*fmkmt1*/*FmKMT1*$^{Ces}$ was used. For wild-type gene amplification FmWT gDNA served as + control. As size marker 1 kb Plus DNA ladder (NEB) was used. **(C)** *FmKMT1* transcript levels in complemented strains. To evaluate suitability of the gained complementation strains, *FmKMT1* transcript levels were assessed by RT-qPCR after 3 days of growth on solid CM. Data represent mean values (n = x).

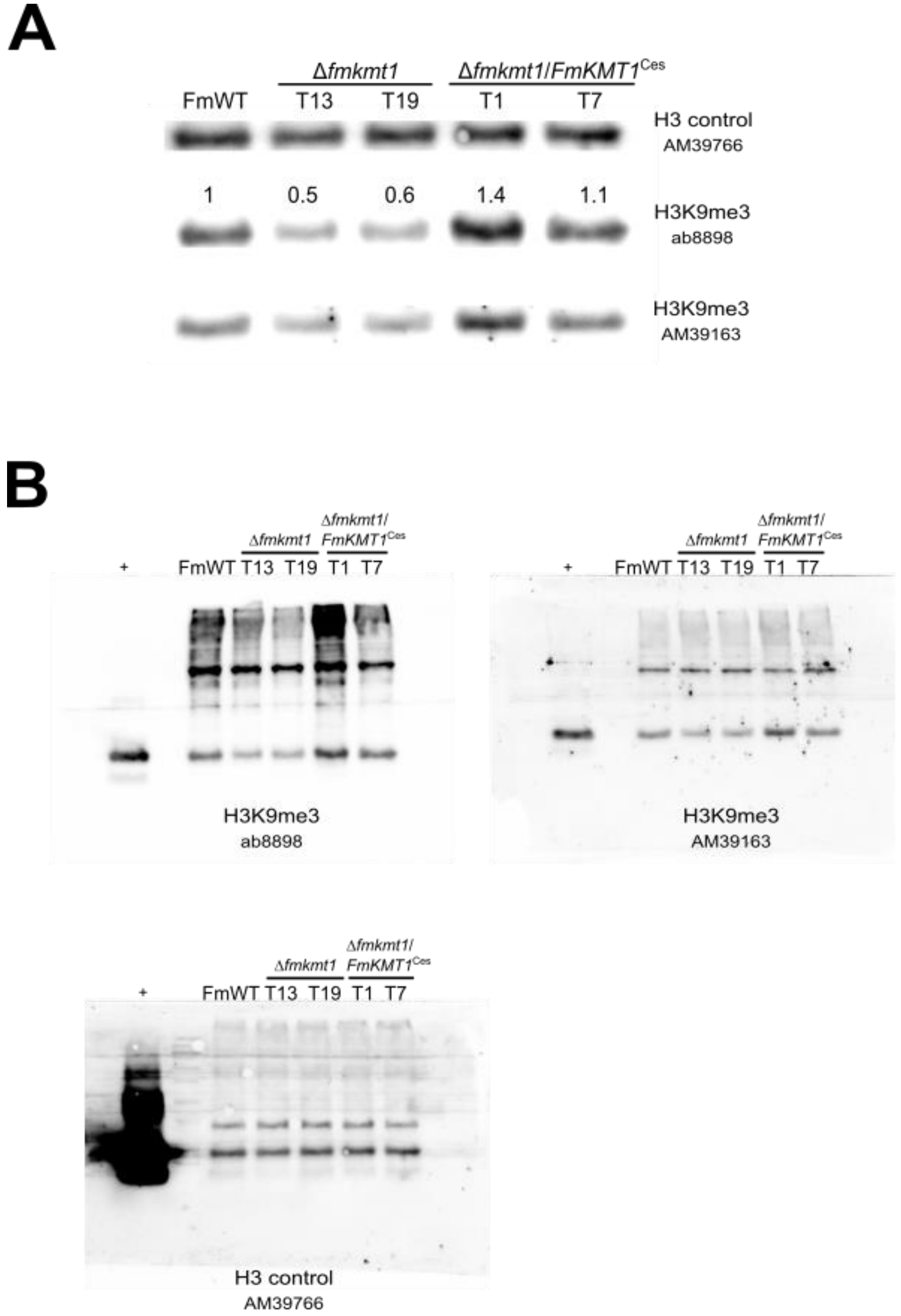


**Figure S3: Lack of *FmKMT1* results in reduced overall H3K9me3 in *Fusarium mangiferae*. (A)** Cropped images of the relevant H3 histone signals as determined by western blotting. For analysis, the *F. mangiferae* wild-type strain (FmWT), the Δ*fmkmt1* and complemented Δ*fmkmt1*/*FmKMT1*$^{Ces}$ strains were grown in liquid ICI medium supplemented with 120 mM $NaNO_3$ for 4 days. Whole proteins were extracted, and 30 µg were separated on a SDS acrylamide gel. Overall H3K9me3 levels were determined using two different anti-H3K9me3 (AB, Abcam; AM, Active Motif), and an anti-H3 C-term-specific antibody was used for referencing. As positive (+) control histones from calf thymus were used. **(B)** Uncropped western blot membranes.

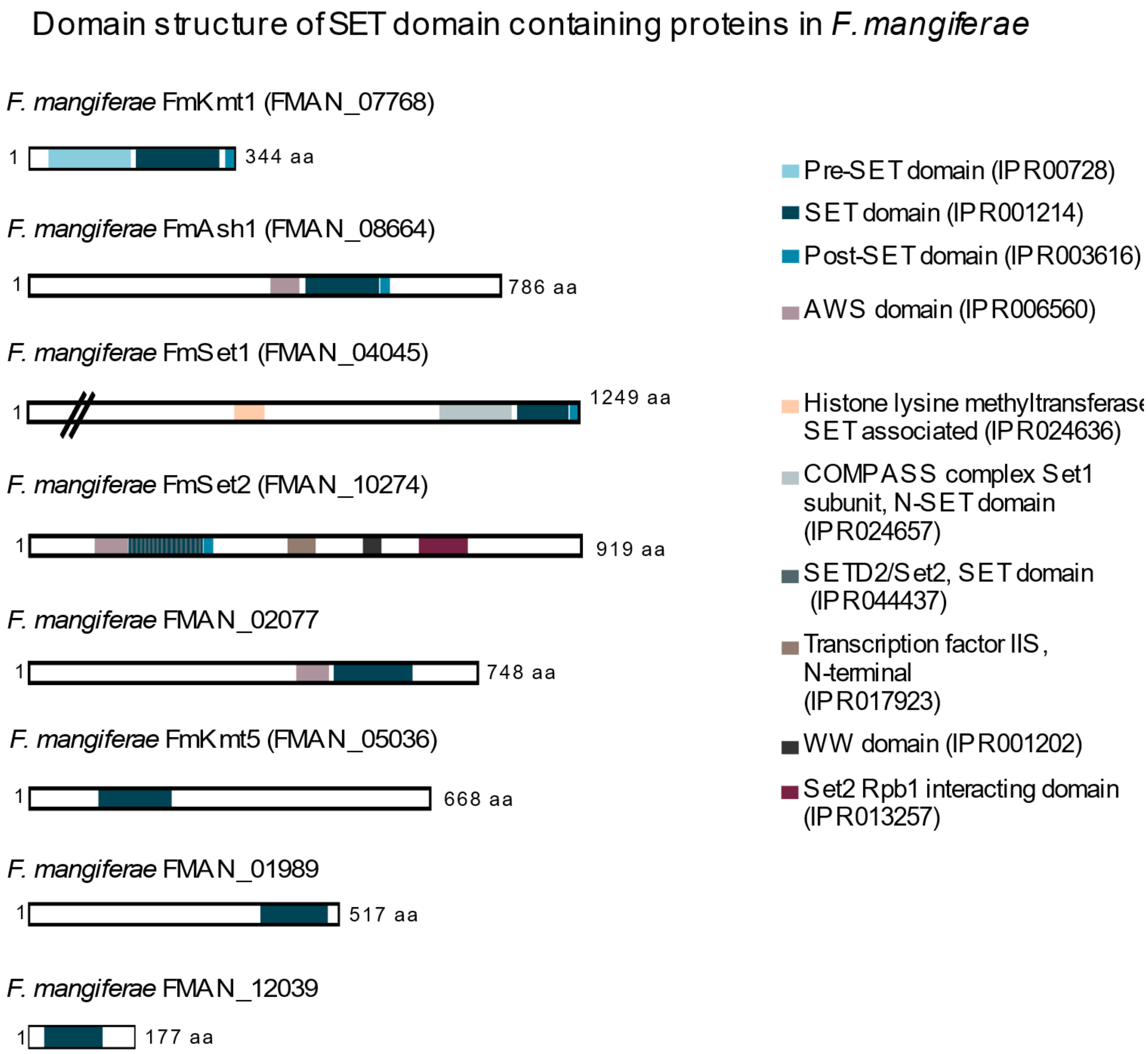


**Figure S4: Domain structures of SET domain containing proteins in *F. mangiferae* (FmWT).** pBLAST search of FmKmt1 SET-domain against FmWT genome. The pBLAST search revealed seven additional SET-domain containing proteins with similarity to FmKmt1 SET-domain. Protein sequences were retrieved from NCBI and subsequently the domain organization was analyzed using InterPro (Blum et al., 2021)**.** The InterPro accsession numbers are shown in the figure legend**.**

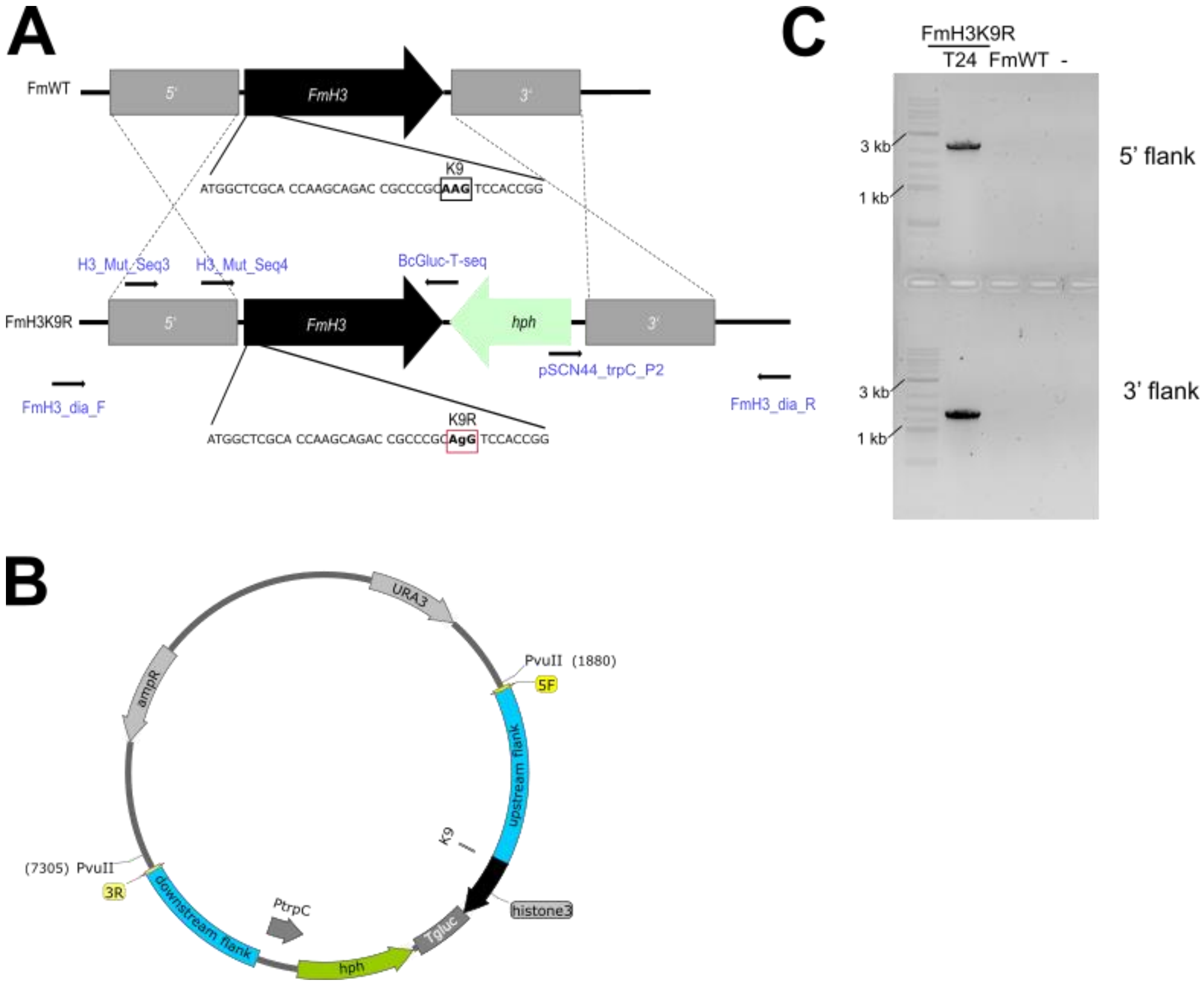


**Figure S5: Strategy, plasmid map and verification of H3K9R mutants by diagnostic PCR and sequencing. (A)** Verification strategy of lysine 9 (K9) to arginine 9 (R9) point mutation. Primer pairs for diagnostic PCRs as well as sequencing are designated in blue. **(B)** FmH3K9R plasmid map. Enzyme used for restriction prior to transformation are shown. **(C)**Verification of successful integration *via* homologous recombination. Homologous integration was verified by the presence of the upstream region (5' flank) with the primer pair FmH3_dia_F//BcGluc-T-seq and the downstream region (3' flank) with FmH3_dia_R//pCSN44_trpC_P2. Successful amino acid exchange was verified by sequencing with the primer pair H3_Mut_Seq3//BcGluc-T-seq for template generation and H3_Mut_Seq4 for sequencing analysis. As size marker 1 kb Plus DNA ladder (NEB) was used. As negative (–) control sterile water was used.

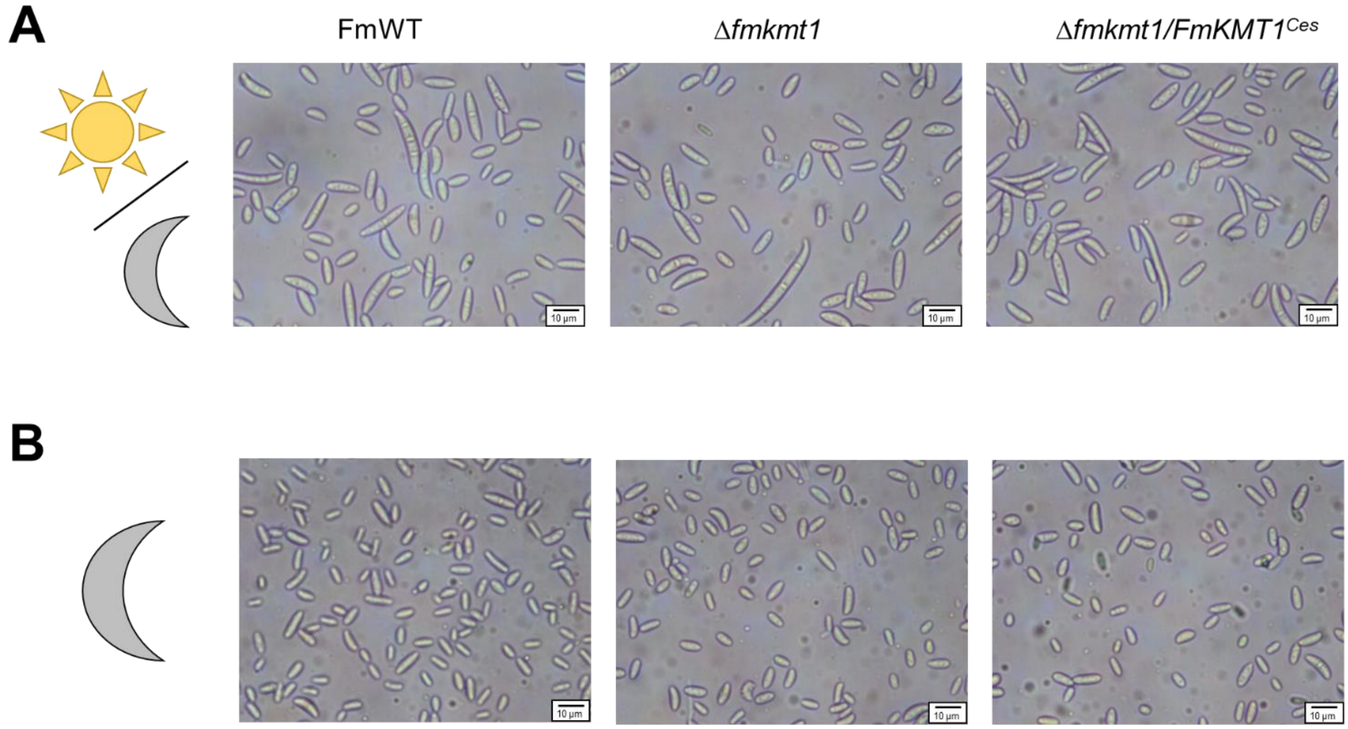


**Figure S6: Conidia production of *F. mangiferae* (FmWT), Δ*fmkmt1* and Δ*fmkmt1*/*FmKMT1*Ces associated mutant strains under light and dark (L/D) and dark (D) conditions.** FmWT, Δ*fmkmt1* and Δ*fmkmt1*/*FmKMT1*Ces were incubated for 7 days on V8 vegetable juice media with **(A)** 16 h light and 8 h dark cycle (L/D), or **(B)** in complete darkness (D). Conidia were observed under light microscope with 40x magnitude. Black bars indicate 10 µm.

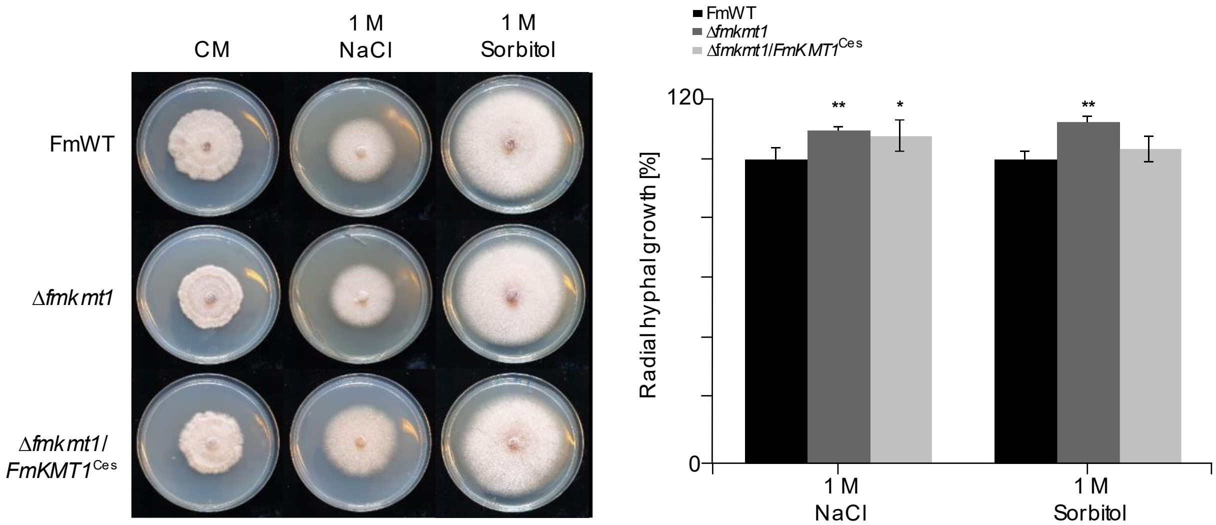


**Figure S7: Involvement of FmKmt1 in osmotic stress response.** Radial hyphal growth of *F. mangiferae* wild-type strain (FmWT), Δ*fmkmt1* and Δ*fmkmt1*/*FmKMT1*$^{Ces}$ on complete medium supplemented with either 1 M NaCl or 1 M sorbitol as compared to unsupplemented CM. Strains were grown for 7 days at 30 °C in the dark. The experiments were performed in technical triplicates and biological duplicates. Hyphal growth of FmWT on the respective media was arbitrarily set to 100%. Growth on media supplemented with stressors was adjusted to normal growth on CM plates of the respective strain. Mean values and standard deviations are given in the diagram. For statistical analysis a student's *t* test was performed. Asterisks above the bars denote significant differences in the radial hyphal growth production of the indicated strains compared to the respective wild-type, * $p<0.05$; ** $p<0.001$.

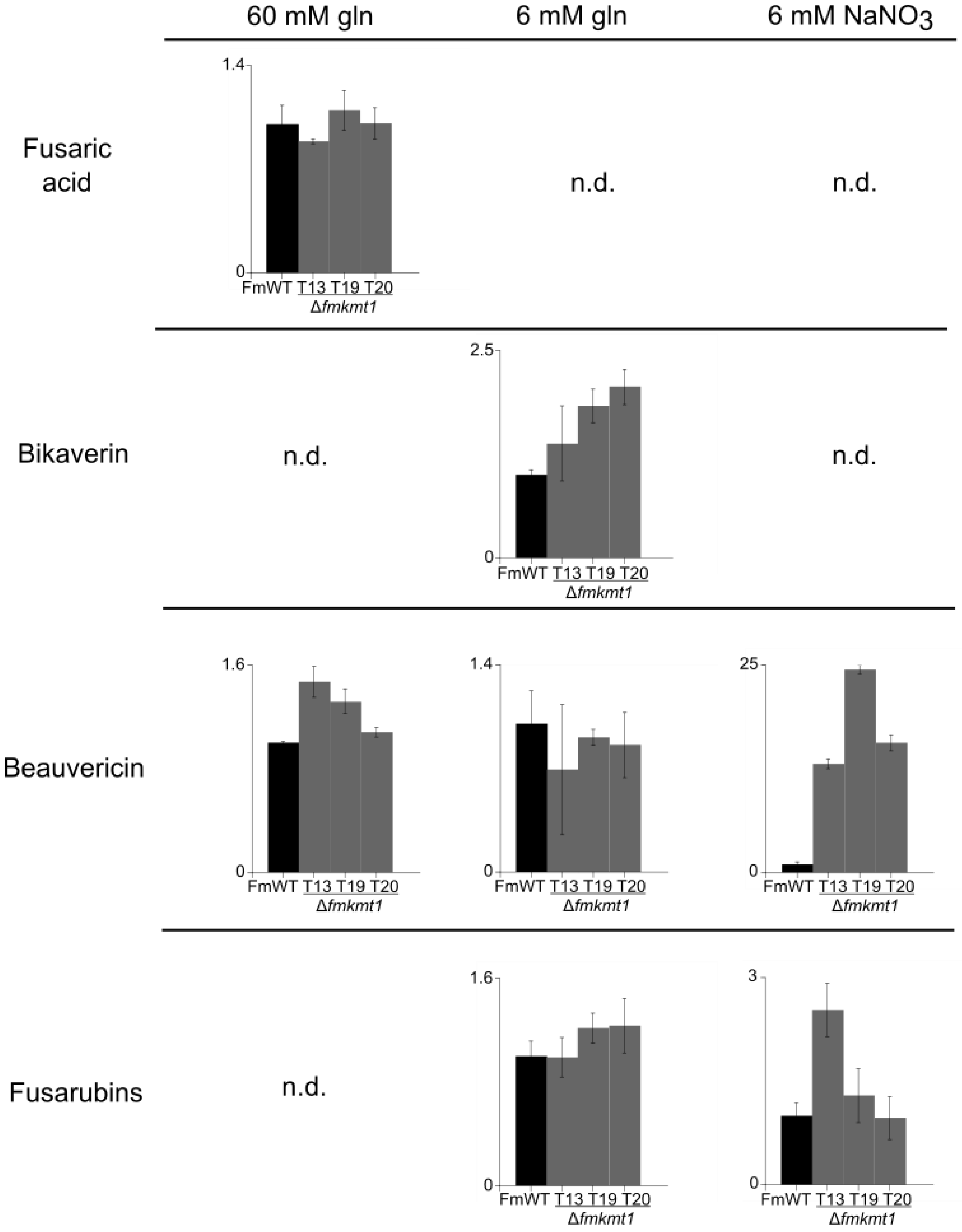
60 mM gln
6 mM gln
6 mM NaNO3
Fusaric acid
Bikaverin
Beauvericin
Fusarubins
n.d.
1.4
2.5
1.6
25
3
0
FmWT T13 T19 T20
Δfmkmt1

**Figure S8: Quantification of known SMs produced by *F. mangiferae* under standard laboratory conditions.** SM production of the *F. mangiferae* wild-type strain (FmWT) and three independent Δ*fmkmt1* mutant strains was quantified using HPLC-HRMS. For this, FmWT and *fmkmt1* deletion strains were cultivated in liquid ICI supplemented with either 60 mM or 6 mM glutamine (gln) and 6 mM $NaNO_3$ for 7 days at 30 °C in constant darkness. Experiments were performed in biological and technical triplicates. Mean values and standard deviations are shown in the diagram. For comparison reasons the wild type was set to 1. n.d., not detected in the supernatant.

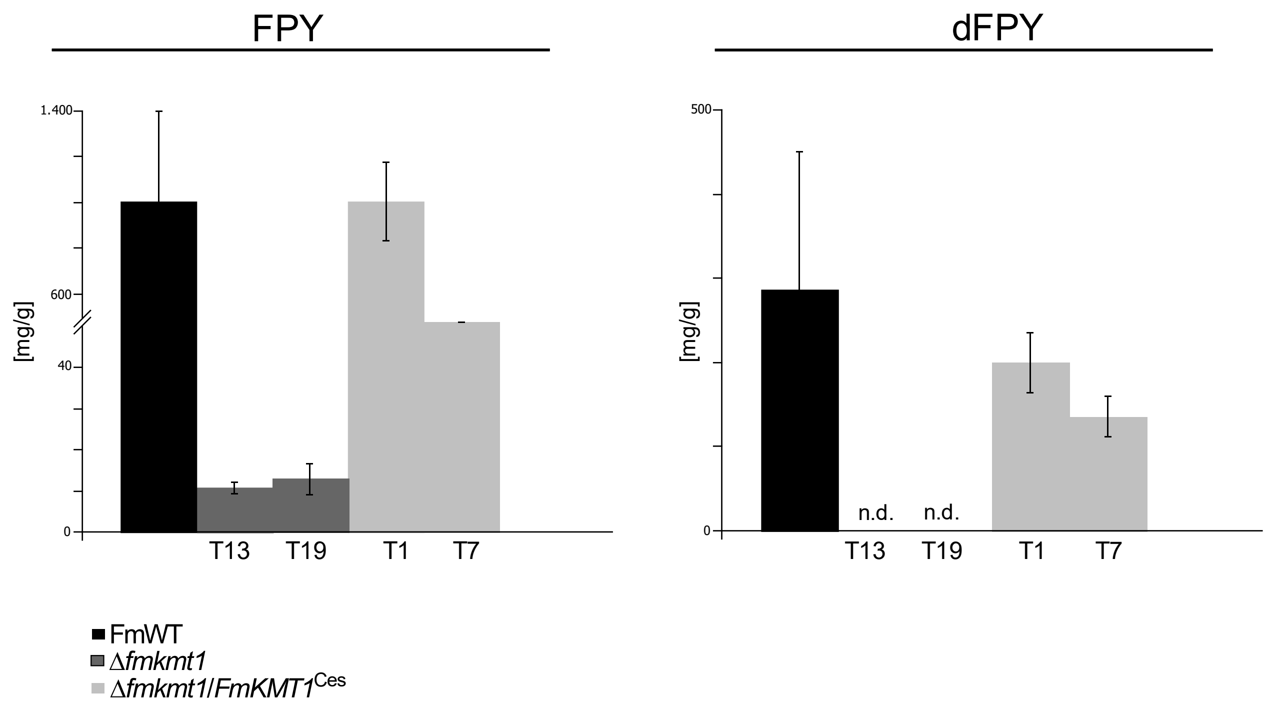


**Figure S9: Quantification of fusapyrone (FPY) and deoxyfusapyrone (dFPY) in *F. mangiferae*.** FPY and dFPY production of the *F. mangiferae* wild-type strain (FmWT), the Δ*fmkmt1* and the Δ*fmkmt1*/*FmKMT1*$^{Ces}$ strains quantified with HPLC-HRMS. For this, indicated fungal strains were grown in liquid ICI supplemented 6 mM $NaNO_3$ for 7 days at 30 °C in constant darkness. The supernatant was directly applied for quantitative analysis. Experiments were performed in in technical triplicates and biological duplicates. Mean values and standard deviations are shown in diagram. n.d., not detected in the supernatant.

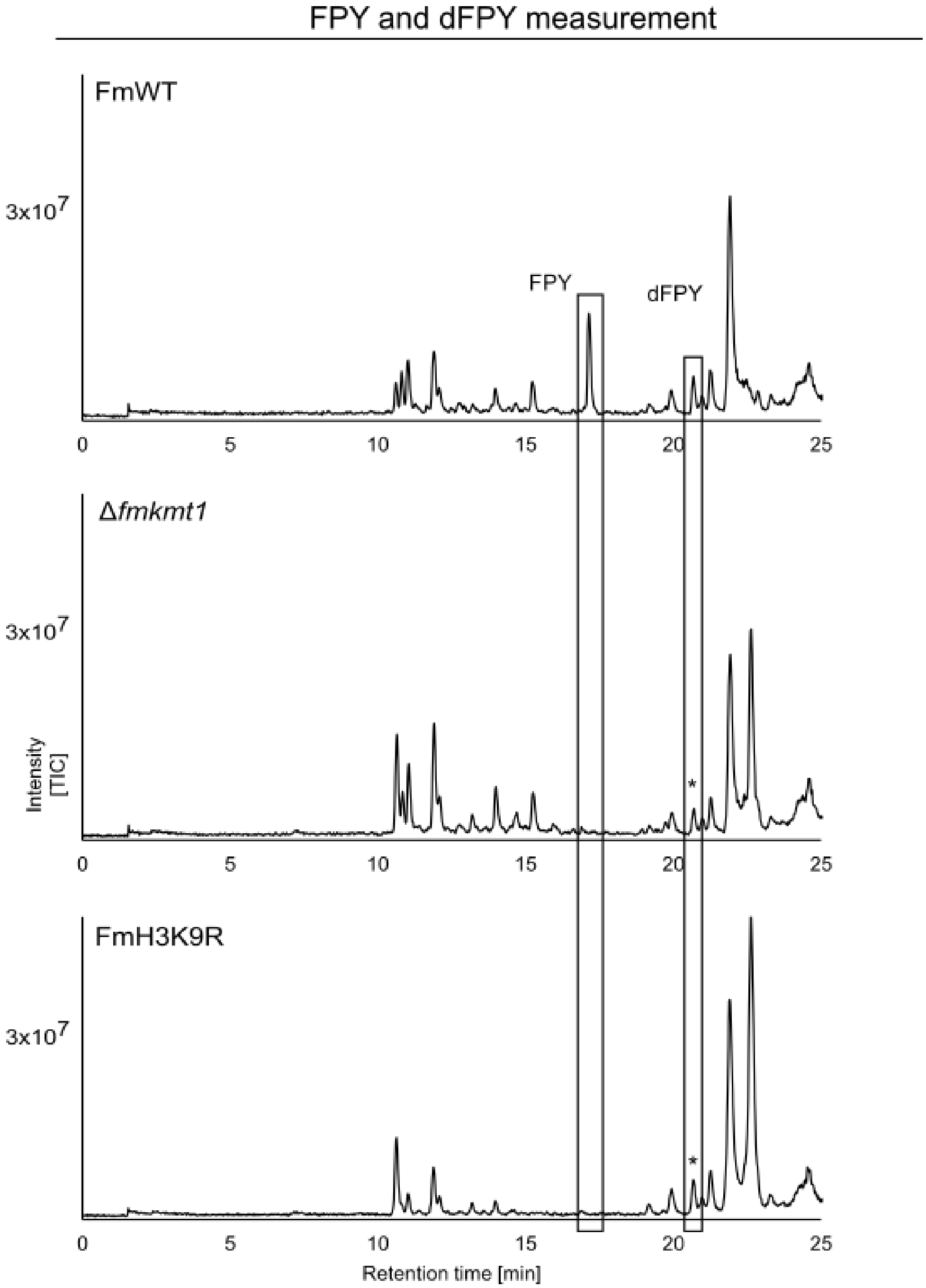


**Figure S10: HPLC-HRMS chromatograms of FPY and dFPY production in the Δ*fmkmt1* deletion and FmH3K9R point mutation strain compared to the wild-type strain (FmWT).** The *F. mangiferae* wild-type strain (FmWT), Δ*fmkmt1* T13 and FmH3K9R T24 were cultivated in FPY-inducing media for 7 days at 30°C. Experiments were performed in technical triplicates. Peaks for FPY and dFPY (boxed) are present in FmWT but absent from Δ*fmkmt1* and FmH3K9R cultures.The asterisk (*) indicates that the observed signal in the Δ*fmkmt1* and FmH3K9R chromatogram with a similar retention time as dFPY is distinct from dFPY, because the signal has a different mass. TIC chromatograms (positive ESI-mode) range from m/z 100-1,000.

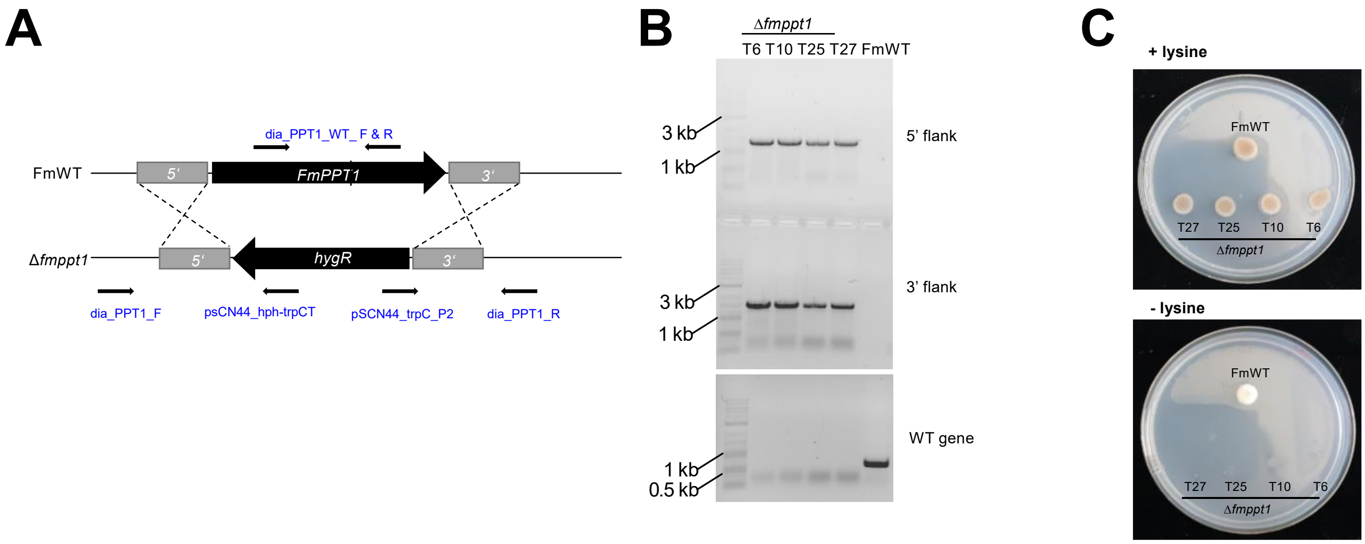


**Figure S11: Strategy and verification of Δ*fmppt1* deletion. (A)** Deletion and verification strategy of Δ*fmppt1* mutants. Primer pairs for diagnostic PCRs are shown in blue. **(B)** Verification of *in situ* deletion of *FmPPT1 via* homologous recombination with the hygR cassette. Homologous integration was verified by the presence of the upstream region (5' flank) with the primers Dia_FmPPT1_F//pCSN44_hph-trpCT and the downstream region (3' flank) by Dia_FmPPT1_R//pCSN44_trpC_P2. Absence of the wild-type gene was verified using the primer pair Dia_WTPPT1_F//R in the case of four independent transformants i.e., Δ*fmppt1* T6, T10, T25 and T27. FmWT gDNA was used as positive control for the wild-type gene, while it served as negative control for correct integration of the resistance cassette. As size marker 1 kb Plus DNA ladder (NEB) was used. **(C)** Lysine auxotrophy test of Δ*fmppt1*. To further verify presence of positive Δ*fmppt1* strains, growth of FmWT and the respective Δ*fmppt1* strains was assessed on synthetic solid media with (+ lysine) and without (- lysine). As expected, Δ*fmppt1* strains did not grow on media without lysine.

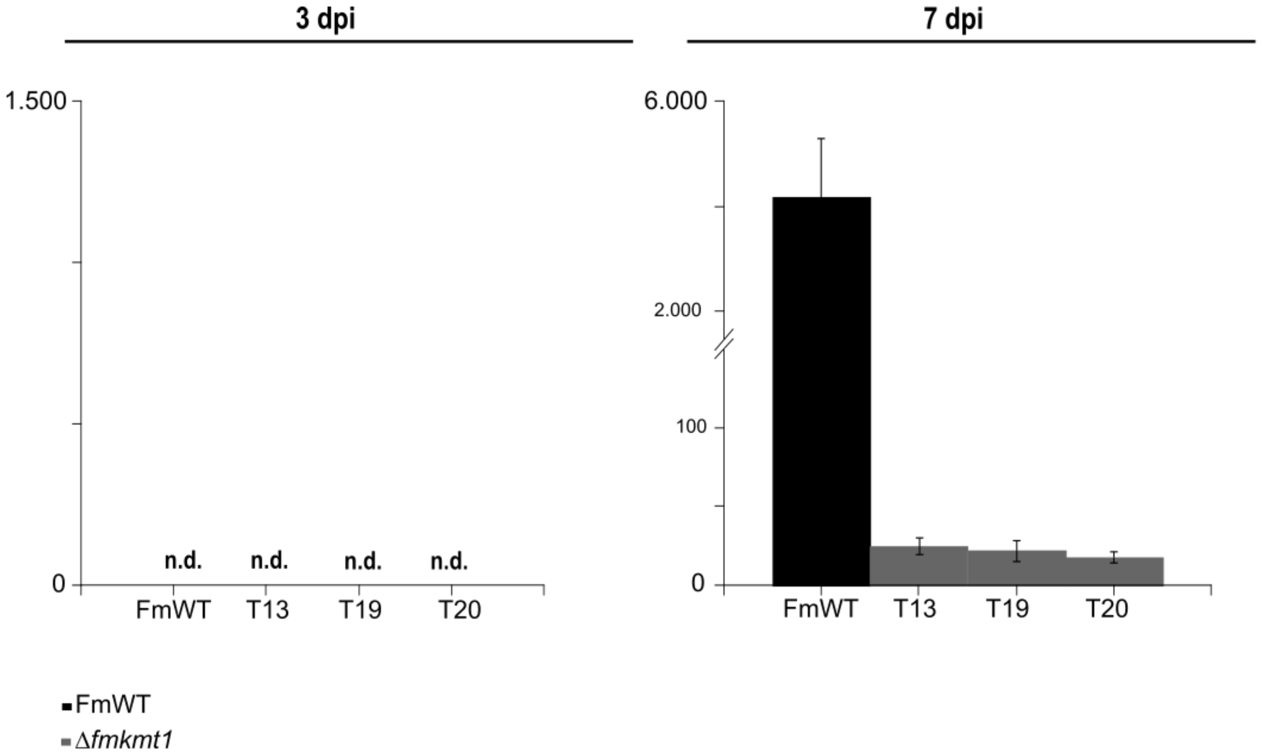


**Figure S12: Quantification of fusapyrone (FPY) and deoxyfusapyrone (dFPY) production in *Fusarium mangiferae* 3 and 7 days post inoculation (dpi).** The *F. mangiferae* wild-type strain (FmWT), and three independent Δ*fmkmt1* mutants were cultivated for 7 days in liquid ICI supplemented with 6 mM $NaNO_3$ at 30 °C. 1 mL aliquots were taken 3 as well as 7 days post inoculation and analyzed *via* HPLC-HRMS. Experiments were performed in technical triplicates and biological duplicates. Mean values and standard deviations are shown in the diagram. n.d., not detected in the supernatant.

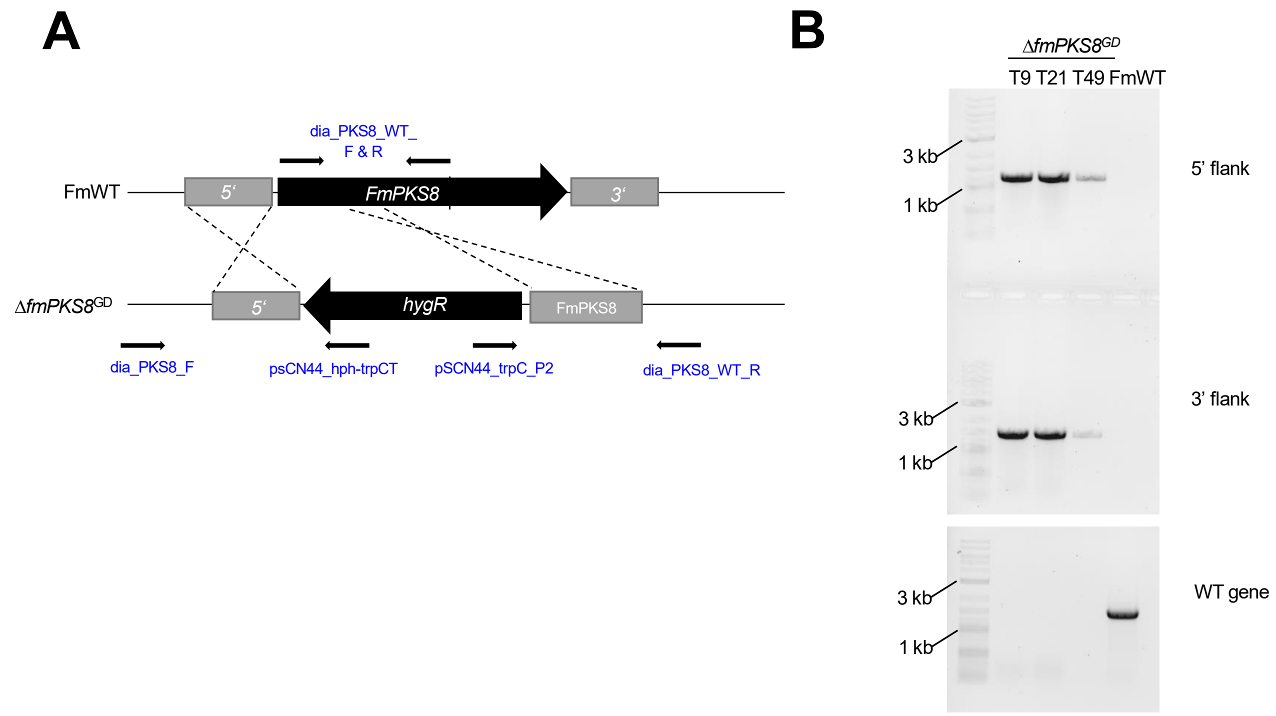


**Figure S13: Strategy and verification of Δ*PKS8* disruption by diagnostic PCR. (A)** Disruption and verification strategy of Δ*fmPKS8* mutants. Primer pairs for diagnostic PCRs are shown in blue. **(B)** Disruption of Δ*fmPKS8 via* homologous recombination of the hygR cassette. Homologous integration was verified by the presence of the upstream region (5' flank) with the primers Dia_FmPKS8_F//pCSN44_hph-trpCT and the downstream region (3' flank) with Dia_FmPKS8_R//pCSN44_trpC_P2. Absence of the wild-type gene was checked by Dia_WTPKS8_F//R for three independent transformants i.e., Δ*fmPKS8* T9, T21 and T49. FmWT gDNA was used as positive control for the wild-type gene, while it served as negative control for correct integration of the resistance cassette. As size marker 1 kb Plus DNA ladder (NEB) was used.

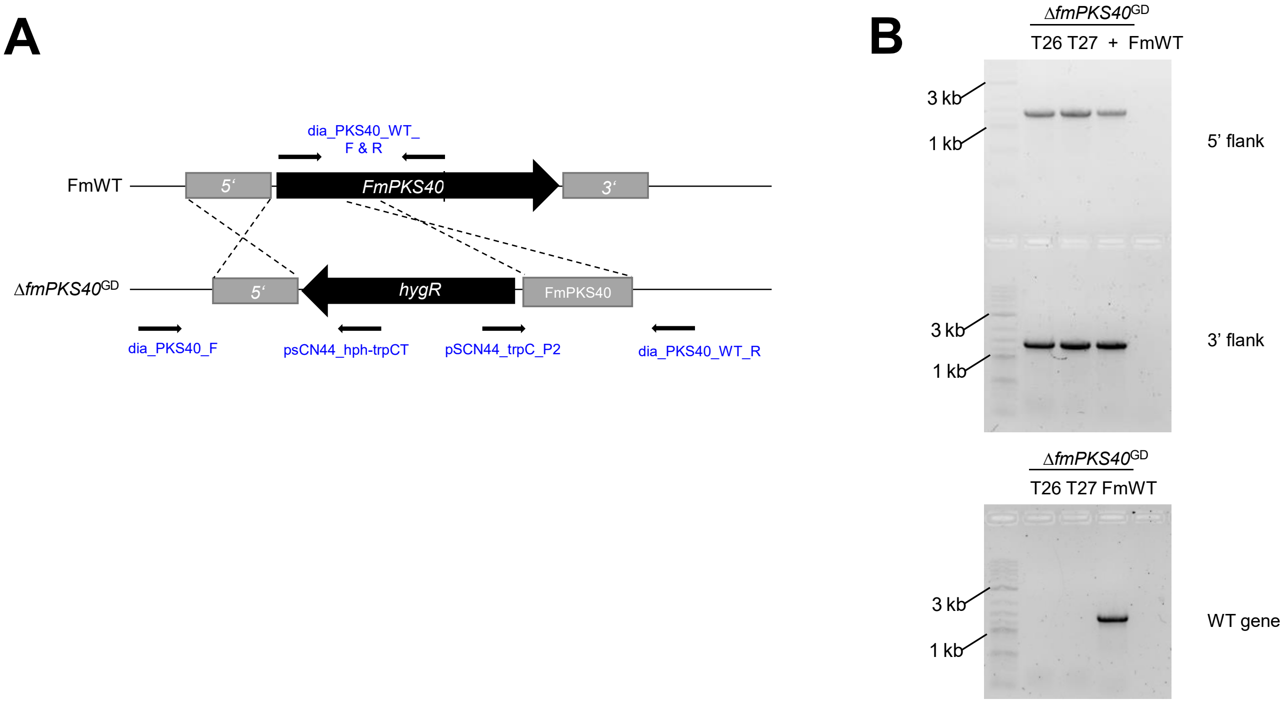


**Figure S14: Strategy and verification of Δ*PKS40* disruption by diagnostic PCR**. **(A)** Disruption strategy and i*n situ* verification of Δ*fmPKS40* mutants. Primer pairs for diagnostic PCRs are depicted in blue. **(B)** Homologous recombination with the hygR cassette was applied for the disruption and generation of Δ*fmPKS40* strains. Homologous integration events were verified by the presence of the upstream region (5' flank) with the primers Dia_PKS40_F//pCSN44_hph-trpCT and the downstream region (3' flank) using Dia_FmPKS40_R//pCSN44_trpC_P2. Absence of the wild-type gene was checked by Dia_WTFmPKS40_F//R for two independent transformants i.e., Δ*fmPKS40* T26 and T27. For 5' and 3' as positive control (+) Δ*fmPKS40* gDNA prior single spore isolation was used, while for wild-type gene diagnostic PCR FmWT gDNA served as positive + control. As size marker 1 kb Plus DNA ladder (NEB) was used.

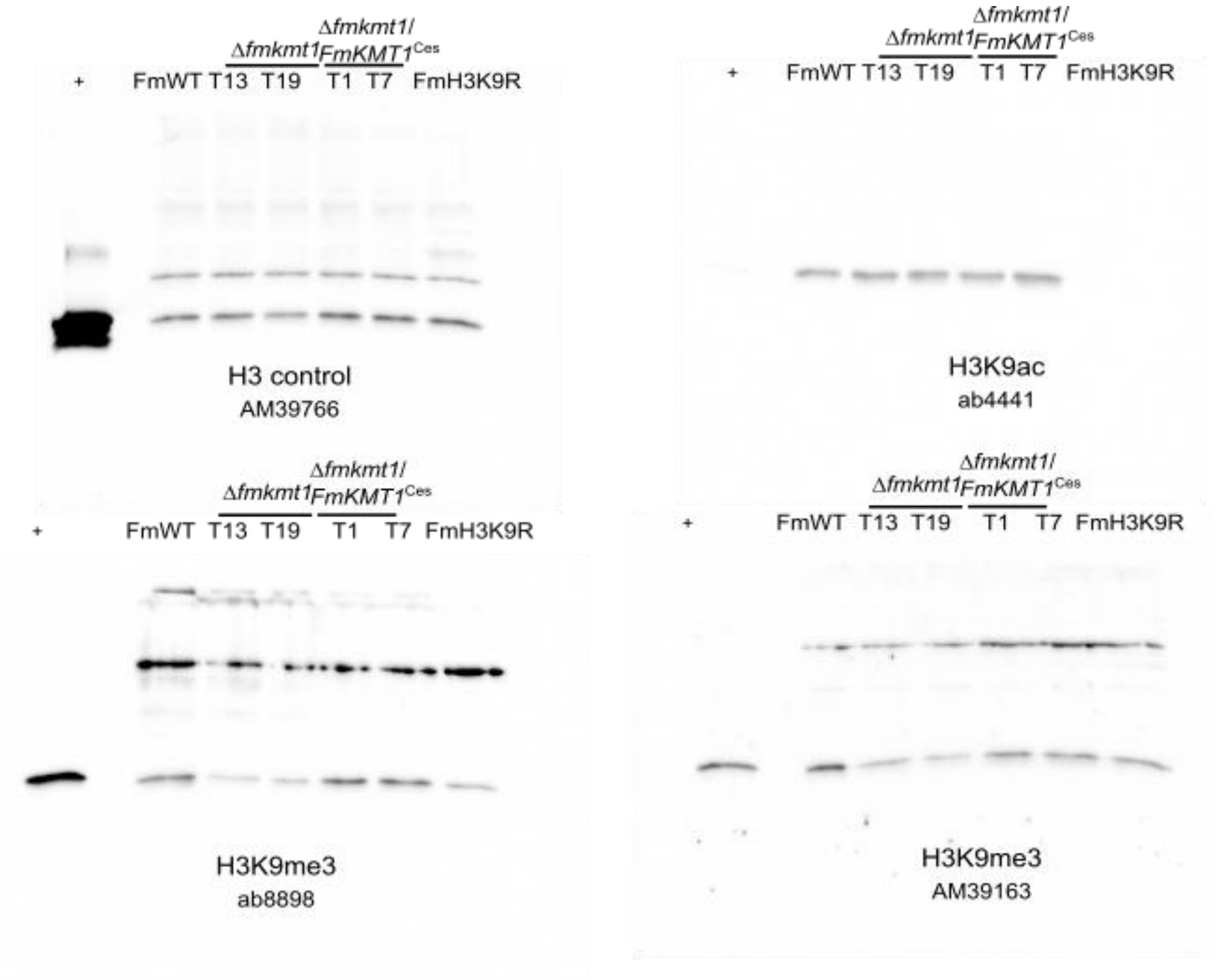


**Figure S15: Loss of FmKmt1 and H3K9R point mutation results in overall reduced H3K9me3 in *Fusarium mangiferae*.** For western blot analysis, the *F. mangiferae* wild-type strain (FmWT), the Δ*fmkmt1*, complemented Δ*fmkmt1*/*FmKMT1*$^{Ces}$ and H3K9R strains were grown in liquid ICI medium supplemented with 120 mM $NaNO_3$ for 4 days. Roughly 50 µg of total proteins were separated on a SDS gel. For determination of overall H3K9me3 levels, two different anti-H3K9me3-specific antibodies (AB, Abcam; AM, Active Motif) were applied. For the quantification of H3K9ac levels an anti-H3K9ac-specific antibody was used, while anti-H3 C-term-specific antibody was used for referencing. As positive (+) control histones from calf thymus were used.

**Table S1: List of primers used in this work.** Introduced overhangs required for yeast recombinational cloning are written in lowercase letters. Primers are designed with * also bind in *F. mangiferae* genome. Primer designed with $^{\alpha}$ bind also in *F. proliferatum* ET1, *F. proliferatum* NRRL62905 and *F. verticillioides* M3125 genomes.

| Gene ID | Primer ID | Primer sequence |
|---|---|---|
| | *Primer used for plasmid generation* | |
| FMAN_07768 | FmKMT1_5F | gccagggttttcccagtcacgacgGGTGATCAAGTTTGAATCTCGG |
| | FmKMT1_5R | ttaacgttactgaaatctccaacCCTCTCTAGTAACACTGCACA |
| | FmKMT1_3F | caatatcatcttctgtctccgacACTTCTTACAAGGGAGGTAGG |
| | FmKMT1_3R | ataacaatttcacacaggaaacagcCCATGGTCATGAAGCTCTTTGC |
| / | FmCIL_tgluc | ATACATCTTATCTACATACGTCACCACAAGAACTTCCTGC |
| / | Geni-Tgluc_R | ACCCCTTCCCCCCAACAAGATATCATCATGCAACATGCATGTACTG |
| / | GeniF | GTCGGAGACAGAAGATGATATTGAAGGAGCCAACAAAACACAGTTCCGACCAC |
| / | Tgluc-nat1R | ATCTTGTTGGGGGGAAGGGGT |
| / | Tgluc_F2 | CGTATGTAGATAAGATGTATG |
| FMAN_09280 | FmPPT1_5F | gccagggttttcccagtcacgacgAGTACAGTAGCAATGCCTCAGCG |
| | FmPPT1_5R | ttaacgttactgaaatctccaacTGCAACTATTGCCTTGGAATGCC |
| | FmPPT1_3F | caatatcatcttctgtctccgacCTCATAGGCTAGTTACATCTCGC |
| | FmPPT1_3R | aacaatttcacacaggaaacagcACTTGCTACCTGCAGAACAAGCC |
| FMAN_15223 | FmPKS8_5F | aacgccagggttttcccagtcacgGGCAAGAACTCAGGTATTGACGG |
| | FmPKS8_5R | tccacttaacgttactgaaatctccCTGGGGTTCCACCACATCTTTACC |

| | | |
|---|---|---|
| | FmPKS8_3F | ccttcaatatcatcttctgtctccgTCTTAGCCCTTTGTCTATGTATCG |
| | FmPKS8_3R | cggataacaatttcacacaggaaacaCCACCAAGTAAGCTCGGTTCTCC |
| | FmPKS8GD_3F | tcaatatcatcttctgtctccgacTCTCATTGAAGAACTCCGTGCTCC |
| | FmPKS8GD_3R | aacaatttcacacaggaaacagcACGAAGGACTTGTTTCCAGTGAACC |
| FMAN_00008 | FmPKS40_5F | gccagggttttcccagtcacgacgGGTCCAGGCTAAATACACAACTGG |
| | FmPKS40_5R | cttaacgttactgaaatctccaacCCGATGAAAACACCAGTGTTTGTTCC |
| | FmPKS40GD_3F | ttcaatatcatcttctgtctccgacCGAGGCTGGAACCAATATATCACC |
| | FmPKS40GD_3R | ataacaatttcacacaggaaacagcCTCATAGAGCTGTCGAAATGAGGTC |
| FMAN_12510 | Histone3_Mut_1F | ccagggttttcccagtcacgacgCACTGGTAGTGTATGGTGATGTGC |
| | Histone3_Mut_2R | catacatcttatctacatacgTTAGTTGCGCTCGCCTCGGAGGC |
| | Histone3_Mut_4F | tcaatAtcatcttctgtcTCCGACGCGATATCACGTATGGGTATTCAGG |
| | Histone3_Mut_4R | actggccgtcgttttacaaGTGAAGAAGACGGTGATCCAGG |
| | Histone3_Mut_K9R_1R | CGGTGGACcTGCGGGCGGTCTGC |
| | Histone3_Mut_K9R_2F | GCAGACCGCCCGCAgGTCCACCG |
| / | Splitmarker_hph_F | CGTTGCAAGACCTGCCTGAA |
| / | Splitmarker_hph_R | GGATGCCTCCGCTCGAAGTA |
| | *Primers for diagnostic PCR* | |
| FMAN_07768 | Dia_FmKMT1_F | TGAATGGTTATAGACGGGAGCC |

| | | |
|---|---|---|
| | Dia_FmKMT1_R | GCAAAGAGCTTCATGACCATGG |
| | Dia_WTKMT1_F | AACGCCATTTCTACTTCCACGG |
| | Dia_WTKMT1_R | TGGGCAGTCTATACTACAGG |
| genR | Gen_seq1 | GAGCCTGAATGTTGAGTGG |
| | Gen_seq3 | CAGCCGATTGTCTGTTGTGC |
| | genR_split_F | GGGAAGGGACTGGCTGCTATTG |
| FMAN_09280 | Dia_FmPPT1_F | GACGAAAAGAAAGACGGAGAGG |
| | Dia_FmPPT1_R | TACCTTGCCCTACATAGTACG |
| | Dia_WTPPT1_F | CAAGACCAAAGACCTCACATCC |
| | Dia_WTPPT1_R | GGCAAACTTGTGCATCTCTAGG |
| FMAN_15223 | Dia_FmPKS8_F | GGCTGGTGAGCATGTTACAATAGG |
| | Dia_FmPKS8_R | CATCGCCCTTAGCATACGAGC |
| | Dia_WTPKS8_F | GGTGTCTTCACAGGTCAAGG |
| | Dia_WTPKS8_R | CCGCAAAGAGGATCTCAGTTCC |
| FMAN_00008 | Dia_FmPKS40_F | AGCCTCACTCTTGGCAAATCC |
| | Dia_WTPKS40_F | CAGACTCTGGTGGTATCTTTGCC |
| | Dia_WTPKS40_R | ACCATGACTTGGTCAATCTCGC |
| / | pCSN44-trpC_P2 | GTGATCCGCCTGGACGACTAAACC |
| / | pCSN44-hph-trpC-T | GGAATAGAGTAGATGCCGACCGG |
| FMAN_12510 | FmH3_dia_R | GCTAGCTGCAGTTTCACACTG |
| | FmH3_dia_F | CACAACGCGTCGAATATGATCC |
| | *Primer used for sequencing* | |

| | | |
|---|---|---|
| FMAN_07768 | Dia_WTFmKMT1_F | AACGCCATTTCTACTTCCACGG |
| | Dia_WTFmKMT1_R | TGGGCAGTCTATACTACAGG |
| / | pRS426_seq | GCCATTCAGGCTGCGCAACTG |
| / | Bcgluc_seq | GGGTCCATGCTAATACTTATGTAC |
| / | Gen_seq1 | GAGCCTGAATGTTGAGTGG |
| / | Gen_seq2 | CTCGTGCTTTACGGTATCGC |
| / | Gen_seq3 | CAGCCGATTGTCTGTTGTGC |
| / | URA3_F | GCTGACATTGGTAATACAGTC |
| / | Tgluc-HiF | CATACGTACATCTGATTTGACAACC |
| / | Hph-F | GTCGGAGACAGAAGATGATATTGAAGGAGC |
| / | Hph-R | GTTGGAGATTTCAGTAACGTTAAGTGGAT |
| / | trpC-P | CCTCCACTAGCTCCAGCCAAGCCC |
| FMAN_12510 | H3_Mut_Seq1 | GGAGAGCTTAAGTACGAGTG |
| | H3_Mut_Seq2 | CTGTGTCATTGAATGAGCC |
| | H3_Mut_Seq3 | GACAGCAAATTCGCCATTGG |
| | H3_Mut_Seq4 | CAACAACAACTATCATCAACC |
| | H3_Mut_Seq5 | TGCTCTCCAGGAGTCCGTCG |
| | H3_Mut_Seq6 | TCAGTCACGCTCTCTAATCC |
| | H3_Mut_Seq7 | GTCATGTGGTCTCCGATCAGG |
| | *Primers for semi-quantitative and RT-qPCR* | |
| | | *cDNA verification* |

| | | |
|---|---|---|
| FFUJ_02611 | cDNA_check_Actin_F | GTATGTGCAAGGCCGGTTTCG |
| | cDNA_check_Actin_R | GAGACCAGGGTACATGGTGG |
| *Housekeeping genes** | | |
| FFUJ_02611 | Actin_F | CCACCATGTACCCTGGTCTCTCC |
| | Actin_R | AATGGAACCACCGATCCAGACGG |
| FFUJ_07385 | ß-TUB_F | GAGGCAGTACGATGGCATGCG |
| | ß-TUB_R | GGTAATCTGCGTCTTCAGCAGCTTCG |
| FFUJ_13490 | GPD_F | GCCTCTGAGGGTGACCTCAAGG |
| | GPD_R | CGTTGTCGTACCAGGAGACCAGC |
| *FmKMT1* | | |
| FMAN_07768 | FmKmt1_F | TGCTAAAGGCCACAGAACGCC |
| | FmKmt1_R | CTGGGAGTGTTGAGTGAGTCGGA |
| *PKS genes* | | |
| FMAN_01974 | FmPKS2_F | GCTGATGGCGGTTGAGTTGAGG |
| | FmPKS2_R | CGAGCTTCGCATCTCAGCGAC |
| FMAN_08330 | FmPKS5_F | GATCCGGGTTTCACCATGCCG |
| | FmPKS5_R | TCTGCTGCTCTATCCAGAGCCG |
| FMAN_15223 | FmPKS8_F | CTCCAGTCCGAGGACGACAGC |
| | FmPKS8_R | ACATTGGTGTCAAGCTCCTCTGGC |
| FMAN_13212 | FmPKS12_F | CACTTACTGCGATTGAGTTGCGGC |
| | FmPKS12_R | CAACGTCTCTCAACGACGGTGC |
| FMAN_15195 | FmPKS17_F | ATGGTGTCGACTCACTGGTTGC |

| | | |
|---|---|---|
| | FmPKS17_R | AATCTCGCTAGCCAACGCTCG |
| FMAN_00008 | FmPKS40_F | TTGATTCCCTCGTCGCAGTCG |
| | FmPKS40_R | AGCCAGTTCAGCCAACTCTTCC |
| FMAN_09800 | FmPKS43_F | TGCTTAAGCCTGTGGAGGACCTTG |
| | FmPKS43_R | CCAAGGACGTCCAGCGTCC |
| FFB14_11413[α] | FfPKS40_F | CCTTGTTGCGGTCGAGTTGC |

**Table S2:** Presence of SM genes and linked products in *F. mangiferae*

| Gene | Gene ID | Product | Reference |
|---|---|---|---|
| **Non-ribosomal peptide synthetases** | | | |
| *FmNRPS1* | FMAN_06841 | malonichrom | (Oide et al., 2014) |
| *FmNRPS2* | FMAN_02774 | ferricrocin (intracellular sideophores) | (Tobiasen et al., 2007; Oide et al., 2014) |
| *FmNRPS3* | FMAN_07066 | | |
| *FmNRPS4* | FMAN_10843 | | |
| *FmNRPS6* | FMAN_13615 | fusarinine (extracellular sideophores) | (Varga et al., 2005) |
| *FmNRPS10* | FMAN_05062 | | |
| *FmNRPS11* | FMAN_13817 | | |
| *FmNRPS12* | FMAN_06437 | | |
| *FmNRPS13* | FMAN_04011 | | |
| *FmNRPS20* | FMAN_06958 | | |
| *FmNRPS21* | FMAN_00090 | | |
| *FmNRPS22* | FMAN_12971 | beauvericin | (Xu et al., 2008; Zhang et al., 2013; Niehaus et al., 2016) |
| *FmNRPS23* | FMAN_15153 | | |
| *FmNRPS24* | FMAN_08295 | | |
| *FmNRPS25* | FMAN_08343 | | |
| *FmNRPS26* | FMAN_08290 | | |
| *FmNRPS34* | FMAN_03675 | fusaric acid | (Brown et al., 2012; Niehaus et al., 2014; Studt et al., 2016) |
| **Polyketide synthases** | | | |
| *FmPKS1/NRPS* | FMAN_03782 | trichosetin | (Hansen et al., 2015) |
| *FmPKS2* | FMAN_01974 | | |
| *FmPKS3* | FMAN_03378 | fusarubins | (Studt et al., 2012) |
| *FmPKS4* | FMAN_06935 | bikaverin | (Linnemannstöns et al., 2002b) |
| *FmPKS5* | FMAN_08330 | | |
| *FmPKS6* | FMAN_03664 | fusaric acid | (Brown et al., 2012; Niehaus et al., 2014; Studt et al., 2016) |
| *FmPKS7* | FMAN_09265 | | |
| *FmPKS8* | FMAN_15223 | | |
| *FmPKS9/NRPS* | FMAN_06335 | | |
| *FmPKS10/NRPS* | FMAN_12219 | fusarin C (pseudogene and abrogated SMGC) | (Brown et al., 2012) |

| | | | |
|---|---|---|---|
| *FmPKS12* | FMAN_13212 | | |
| *FmPKS13* | FMAN_15141 | gibepyrones | (Janevska et al., 2016) |
| *FmPKS14* | FMAN_13912 | | |
| *FmPKS17* | FMAN_15195 | | |
| *FmPKS18* | FMAN_15205 | | |
| *FmPKS20/NRPS* | FMAN_11871 | | |
| *FmPKS38* | FMAN_02337 | | |
| *FmPKS40* | FMAN_00008 | fusapyrone / deoxyfusapyrone | **This study** |
| *FmPKS43* | FMAN_09800 | | |
| **Dimethylallytryptophane synthases** | | | |
| *FmDMATS1* | FMAN_13061 | r-*N*-DMAT | (Arndt et al., 2017) |
| *FmDMATS2* | FMAN_06887 | | |
| *FmDMATS3* | FMAN_06328 | | |
| *FmDMATS4* | FMAN_06531 | | |
| **Terpene cyclases** | | | |
| *FmDTC1-1* | FMAN_08250 | gibberellins | (Tudzynski and Hölter, 1998) |
| *FmTrTC1* | FMAN_05579 | | |
| *FmTeTC1* | FMAN_14945 | phytoene | (Linnemannstöns et al., 2002a) |
| *FmSTC1* | FMAN_02036 | | |
| *FmSTC2* | FMAN_01129 | | |
| *FmSTC3* | FMAN_03293 | (+)-eremophilene | (Burkhardt et al., 2016) |
| *FmSTC4* | FMAN_13218 | (+)-koraiol | (Brock et al., 2013) |
| *FmSTC5* | FMAN_14887 | (–)-guaia-6,10(14)-diene | (Burkhardt et al., 2016) |
| *FmSTC6* | FMAN_11750 | (–)-α-acorenol | (Brock et al., 2013) |
| *FmSTC7* | FMAN_16004 | | |
| *FmSTC8* | FMAN_12837 | | |
| *FmSTC9* | FMAN_06479 | | |

**Table 3:** RT-qPCR raw data from FPY SMGC co-expression studies

| | Gene ID | 60 mM glutamine | | 6 mM glutamine | | 120 mM $NaNO_3$ | | 6 mM $NaNO_3$ | |
|---|---|---|---|---|---|---|---|---|---|
| **Housekeeping genes** | | | | | | | | | |
| Actin | FMAN_04166 | / | 21.34 | 21.52 | 21.88 | 20.41 | 20.40 | 18.02 | 18.03 |
| Tubulin | FMAN_05925 | 26.50 | 26.12 | 26.54 | 26.59 | 24.38 | 24.53 | 23.12 | 23.14 |
| GPD | FMAN_07563 | 19.60 | 19.90 | 20.53 | 20.72 | 18.94 | 19.07 | 17.37 | 17.41 |
| **Investigated genes/ Putative cluster genes** | | | | | | | | | |
| | FMAN_00001 | 22.43 | 22.33 | 23.08 | 22.90 | 22.17 | 22.15 | 20.17 | 20.14 |
| | FMAN_00002 | 35.20 | 36.36 | 24.52 | 24.63 | 33.08 | 34.31 | 17.52 | 17.46 |
| | FMAN_00003 | 36.99 | 36.96 | 26.22 | 26.55 | 36.25 | 36.31 | 19.31 | 19.44 |
| | FMAN_00004 | 28.14 | 28.34 | 25.07 | 25.08 | 28.06 | 28.11 | 18.15 | 18.13 |
| | FMAN_00005 | / | / | 27.60 | 27.77 | 39.50 | / | 20.81 | 20.70 |
| | FMAN_00006 | 36.55 | 38.33 | 24.29 | 24.49 | 37.30 | / | 18.32 | 17.64 |
| | FMAN_00007 | 39.81 | / | 27.03 | 27.38 | / | 34.84 | 18.32 | 18.52 |
| | FMAN_00008 | 36.98 | 38.26 | 25.40 | 25.26 | 37.60 | 36.34 | 17.60 | 17.50 |
| | FMAN_00009 | 36.82 | 33.73 | 35.19 | 33.37 | 36.53 | 34.44 | 31.27 | 31.23 |
| | FMAN_000010 | 31.21 | 31.39 | 31.18 | 31.09 | 32.55 | 32.32 | 29.19 | / |
| | FMAN_000011 | 27.30 | 27.24 | 32.81 | 32.61 | 28.27 | 28.58 | 29.31 | 29.60 |
| | FMAN_000012 | 33.75 | 32.42 | 33.37 | 32.93 | 32.76 | 32.55 | 32.03 | 32.52 |
| | FMAN_000013 | 29.51 | 29.51 | 34.34 | 33.65 | 30.20 | 30.51 | 32.05 | 32.70 |

**Table 4:** Detailed analysis of putative *fmfpy* SMGC

| Gene ID | Length (bp) | Number of amino acids | InterPro annotation | Family type (F),<br>Domains (D) and motifs (M) | Predicted function |
|---|---|---|---|---|---|
| *FmFPY1* (FMAN_00002) | 1666 | 519 | IPR001128<br>IPR002402<br>IPR002974 | Cytochrome P450 (F)<br>Cytochrome P450, E-class, group II (F)<br>Cytochrome P450, E-class, CYP52 (F) | Related to cytochrome P450 alkane hydroxylase |
| *FmFPY2* (FMAN_00003) | 1190 | 349 | IPR000683<br>IPR004104 | Oxidoreductase, N-terminal (D)<br>Gfo/Idh/MocA-like oxidoreductase, C-terminal (D) | Related to dehydrogenases and related proteins |
| *FmFPY3* (FMAN_00004) | 2157 | 597 | IPR011701<br>IPR020846 | Major facilitator superfamily (F)<br>Major facilitator superfamily domain (D) | Related to multidrug transporter |
| *FmFPY4* (FMAN_00005) | 1110 | 369 | | | FAD dependent oxidoreductase |
| *FmFPY5* (FMAN_00006) | 834 | 218 | IPR005645 | Serine hydrolase FSH (D) | FSH1 domain-containing protein |
| *FmFPY6* (FMAN_00007) | 1696 | 546 | IPR002213 | UDP-glucuronosyl/UDP-glucosyltransferase (F) | Related to UDP-glucosyl transferase family protein |
| *FmFPY7* (FMAN_00008) | 7580 | 2474 | IPR009081<br>IPR013149<br>IPR013217<br>IPR013968<br>IPR014030<br>IPR014031<br>IPR014043<br>IPR020806 | Phosphopantetheine binding ACP domain (D)<br>Alcohol dehydrogenase, C-terminal (D)<br>Methyltransferase type 12<br>Polyketide synthase, ketoreductase domain (D)<br>Beta-ketoacyl synthase, N-terminal (D)<br>Beta-ketoacyl synthase, C-terminal (D)<br>Acyl transferase<br>Polyketide synthase, phosphopantetheine-binding | FmPKS40 |

| | |
|---|---|
| IPR020807 | domain (D) |
| IPR020841 | Polyketide synthase, dehydratase domain |
| IPR020843 | Polyketide synthase, beta-ketoacyl synthase domain (D) |
| IPR032821 | Polyketide synthase, enoylreductase domain (D) |
| | Polyketide synthase, C-terminal extension (D) |

**Table 5: Comparison of *FPY* SMBGC with other members of the FFC**

| Predicted gene function | *F. mangiferae* MRC7560 | *F. fujikuroi* B14 | *F. proliferatum* *ET1* | *F. proliferatum* NRRL62905 | *F. verticillioides* M3125 |
|---|---|---|---|---|---|
| Unknown | FMAN_00001 | FFB14_11405 | / | FPRN_00001 | / |
| Cytochrome P450 alkane hydroxylase | **FMAN_00002** | **FFB14_11407** | **FPRO_00007** | / | / |
| Dehydrogenase | **FMAN_00003** | **FFB14_11408** | **FPRO_00008** | / | / |
| Multidrug transporter | **FMAN_00004** | **FFB14_11409** | **FPRO_00009** | / | **FVEG_09964** |
| **FAD dependent oxidoreductase** | **FMAN_00005** | **FFB14_11410** | **FPRO_00010** | / | **FVEG_09963** |
| **FSH1 domain-containing protein** | **FMAN_00006** | **FFB14_11411** | **FPRO_00011** | / | / |
| **UDP-glucosyl transferase family protein** | **FMAN_00007** | **FFB14_11412** | **FPRO_00012** | / | / |
| **PKS40** | **FMAN_00008** | **FFB14_11413** | **FPRO_00013** | **FPRN_00002** | **FVEG_16698T0** |
| Cellulose binding protein | FMAN_00009 | / | / | / | / |
| Mfs-multidrug-resistance transporter | FMAN_00010 | FFB14_11414 | FPRO_00014 | FPRN_00003 | FVEG_09958 |
| Unknown | FMAN_00011 | FFB14_11415 | FPRO_00015 | FPRN_00004 | / |
| Transporter | FMAN_00012 | FFB14_11416 | FPRO_00016 | FPRN_00005 | / |
| Zn(2)-C6 fungal-type domain-containing protein | FMAN_00013 | FFB14_11417 | FPRO_00017 | FPRN_00006 | / |

## References

Arndt, B., Janevska, S., Schmid, R., Hübner, F., Tudzynski, B., and Humpf, H.-U. (2017). A Fungal N-Dimethylallyltryptophan Metabolite from Fusarium fujikuroi. *ChemBioChem* 18, 899–904. doi:10.1002/cbic.201600691.

Blum, M., Chang, H. Y., Chuguransky, S., Grego, T., Kandasaamy, S., Mitchell, A., et al. (2021). The InterPro protein families and domains database: 20 years on. *Nucleic acids research* 49, D344–D354. doi:10.1093/nar/gkaa977.

Brock, N. L., Huss, K., Tudzynski, B., and Dickschat, J. S. (2013). Genetic Dissection of Sesquiterpene Biosynthesis by Fusarium fujikuroi . *ChemBioChem* 14, 311–315. doi:10.1002/cbic.201200695.

Brown, D. W., Butchko, R. A. E., Busman, M., and Proctor, R. H. (2012). Identification of gene clusters associated with fusaric acid, fusarin, and perithecial pigment production in Fusarium verticillioides. *Fungal Genetics and Biology* 49, 521–532. doi:10.1016/j.fgb.2012.05.010.

Burkhardt, I., Siemon, T., Henrot, M., Studt, L., Rösler, S., Tudzynski, B., et al. (2016). Mechanistic Characterisation of Two Sesquiterpene Cyclases from the Plant Pathogenic Fungus Fusarium fujikuroi. *Angewandte Chemie* 128, 8890–8893. doi:10.1002/ange.201603782.

Hansen, F. T., Gardiner, D. M., Lysøe, E., Fuertes, P. R., Tudzynski, B., Wiemann, P., et al. (2015). An update to polyketide synthase and non-ribosomal synthetase genes and nomenclature in Fusarium. *Fungal Genetics and Biology* 75, 20–29. doi:10.1016/j.fgb.2014.12.004.

Janevska, S., Arndt, B., Niehaus, E. M., Burkhardt, I., Rösler, S. M., Brock, N. L., et al. (2016). Gibepyrone biosynthesis in the rice pathogen Fusarium fujikuroi is facilitated by a small polyketide synthase gene cluster. *Journal of Biological Chemistry* 291, 27403–27420. doi:10.1074/jbc.M116.753053.

Linnemannstöns, P., Prado, M. M., Fernández-Martín, R., Tudzynski, B., and Avalos, J. (2002a). A carotenoid biosynthesis gene cluster in Fusarium fujikuroi: The genes carB and carRA. *Molecular Genetics and Genomics* 267, 593–602. doi:10.1007/s00438-002-0690-5.

Linnemannstöns, P., Schulte, J., del Mar Prado, M., Proctor, R. H., Avalos, J., and Tudzynski, B. (2002b). The polyketide synthase gene pks4 from Gibberella fujikuroi encodes a key enzyme in the biosynthesis of the red pigment bikaverin. *Fungal Genetics and Biology* 37, 134–148. Available at: www.academicpress.com.

Niehaus, E. M., von Bargen, K. W., Espino, J. J., Pfannmüller, A., Humpf, H. U., and Tudzynski, B. (2014). Characterization of the fusaric acid gene cluster in Fusarium fujikuroi. *Applied Microbiology and Biotechnology* 98, 1749–1762. doi:10.1007/s00253-013-5453-1.

Niehaus, E.-M., Studt, L., von Bargen, K. W., Kummer, W., Humpf, H.-U., Reuter, G., et al. (2016). Sound of silence: The beauvericin cluster in Fusarium fujikuroi is controlled by cluster-specific and global regulators mediated by H3K27 modification. *Environmental Microbiology* 18, 4282–4302. doi:10.1111/1462-2920.13576.

Oide, S., Berthiller, F., Wiesenberger, G., Adam, G., and Turgeon, B. G. (2014). Individual and combined roles of malonichrome, ferricrocin, and TAFC siderophores in Fusarium graminearum pathogenic and sexual development. *Frontiers in Microbiology* 5. doi:10.3389/fmicb.2014.00759.

Studt, L., Janevska, S., Niehaus, E. M., Burkhardt, I., Arndt, B., Sieber, C. M. K., et al. (2016). Two separate key enzymes and two pathway-specific transcription factors are involved in fusaric acid biosynthesis in Fusarium fujikuroi. *Environmental Microbiology* 18, 936–956. doi:10.1111/1462-2920.13150.

Studt, L., Wiemann, P., Kleigrewe, K., Humpf, H., and Tudzynski, B. (2012). Biosynthesis of Fusarubins Accounts for Pigmentation of Fusarium fujikuroi Perithecia. *Applied and Environmental Microbiology* 78, 4468–4480. doi:10.1128/AEM.00823-12.

Tobiasen, C., Aahman, J., Ravnholt, K. S., Bjerrum, M. J., Grell, M. N., and Giese, H. (2007). Nonribosomal peptide synthetase (NPS) genes in Fusarium graminearum, F. culmorum and F. pseudograminearium and identification of NPS2 as the producer of ferricrocin. *Current Genetics* 51, 43–58. doi:10.1007/s00294-006-0103-0.

Tudzynski, B., and Hölter, K. (1998). Gibberellin Biosynthetic Pathway in Gibberella fujikuroi: Evidence for a Gene Cluster. *Fungal Genetics and Biology* 25, 157–170.

Varga, J., Kocsube, S., Toth, B., and Mesterhazy, A. (2005). NONRIBOSOMAL PEPTIDE SYNTHETASE GENES IN THE GENOME OF FUSARIUM GRAMINEARUM, CAUSATIVE AGENT OF WHEAT HEAD BLIGHT. *Acta Biologica Hungarica* 56, 375–388.

Xu, Y., Orozco, R., Wijeratne, E. M. K., Gunatilaka, A. A. L., Stock, S. P., and Molnár, I. (2008). Biosynthesis of the Cyclooligomer Depsipeptide Beauvericin, a Virulence Factor of the Entomopathogenic Fungus Beauveria bassiana. *Chemistry and Biology* 15, 898–907. doi:10.1016/j.chembiol.2008.07.011.

Zhang, T., Zhuo, Y., Jia, X. P., Liu, J. T., Gao, H., Song, F. H., et al. (2013). Cloning and characterization of the gene cluster required for beauvericin biosynthesis in Fusarium proliferatum. *Science China Life Sciences* 56, 628–637. doi:10.1007/s11427-013-4505-1.
